# Optimizing decision-making with aggregation operators for generalized intuitionistic fuzzy sets and their applications in the tech industry

**DOI:** 10.1038/s41598-024-57461-9

**Published:** 2024-07-17

**Authors:** Muhammad Wasim, Awais Yousaf, Hanan Alolaiyan, Muhammad Azeem Akbar, Alhanouf Alburaikan, Hamiden Abd El-Wahed Khalifa

**Affiliations:** 1 Department of Mathematics, The Islamia University of Bahawalpur, Bahawalpur, 63100 Pakistan; 2 Department of Mathematics, King Saud University, 12271 Riyadh, Saudi Arabia; 3 Software Engineering Department, Lappeenranta-Lahti University of Technology, 53851 Lappeenranta, Finland; 4 Department of Mathematics, College of Science and Arts, Qassim University, 51951 Al-Badaya, Saudi Arabia; 5 Department of Operations and Management Research, Faculty of Graduate Studies for Statistical Research, Cairo University, Giza, 12613 Egypt

**Keywords:** Decision-making and optimization, Intuitionistic fuzzy set, Generalized intuitionistic fuzzy set, Score function, Aggregation operators, Computational science, Information technology, Pure mathematics, Scientific data, Statistics

## Abstract

Intuitionistic fuzzy sets (IFSs) represent a significant advancement in classical fuzzy set (FS) theory. This study advances IFS theory to generalized intuitionistic fuzzy sets (GIFS_B_s) and introduces novel operators GIFWAA, GIFWGA, GIFOWAA, and GIFOWGA, tailored for GIFS_B_s. The primary aim is to enhance decision-making capabilities by introducing aggregation operators within the GIFS_B_ framework that align with preferences for optimal outcomes. The article introduces new operators for GIFS_B_s characterized by attributes like idempotency, boundedness, monotonicity and commutativity, resulting in aggregated values aligned with GIFNs. A comprehensive analysis of the relationships among these operations is conducted, offering a thorough understanding of their applicability. These operators are practically demonstrated in a multiple-criteria decision-making process for evaluating startup success in the Tech Industry.

## Introduction

Intuitionistic Fuzzy Sets (IFSs) represent a significant advancement in classical Fuzzy Set (FS) theory. Zadeh^1^ introduced the theory of fuzzy sets (FS) in 1965, laying the foundation for a novel approach to handling data uncertainty. Building on this seminal work, Atanassov^2^ extended the theory in 1986 with the introduction of IFSs, which have since proven to be a potent tool for effectively addressing data uncertainties. Intuitionistic Fuzzy Sets (IFSs) offer a unique approach to representing elements with dual-degree attributes, building upon Zadeh's foundational fuzzy set theory. IFSs encapsulate both membership and non-membership degrees, ensuring that their combined sum is equal to unity. The emergence of IFSs has generated significant academic attention across various fields. The indispensability of their effectiveness in traversing complex decision-making settings has made them invaluable for the management of uncertainty. Scholars have extensively investigated multiple aspects within the Intuitionistic Fuzzy Sets (IFS) framework. The relationship between IFSs has been established by similarity measurements, as proposed by Hung and Yang in 2004, using the Hausdorff distance.^3^ The nonlinear programming model proposed by Garg and Arora^4^ (2017) highlighted the potential use of Interval-Valued Intuitionistic Fuzzy Soft Sets (IFSs) in decision-making processes. Expanding upon the aforementioned groundwork, Garg^5^ further developed these contributions, highlighting the efficacy of employing a generalized entropy measure in the context of Intuitionistic Fuzzy Sets (IFS). The exploration of similarity measures and aggregation operators has been a prominent area of research in decision-making scenarios employing IFS. The operators, namely the ordered weighted geometric and hybrid geometric aggregation, were first introduced by Xu and Chen^6^ and Wei and Wang^7^ in 2007. The operators such as IFWA, IFWG, IFOWA, and IFOWG were introduced by Xu and Yager^8^ and^9^ in 2006 and 2007. In addition to aggregation operators, researchers have extensively examined the characteristics and interconnections of other operations inside IFSs. Significant contributions have been made by Atanassov in 1986^2^, Atanassov in 1994^10^, De et al. in 1986^11^, Riecan^12^, Cuong et al.^13^, and Parvathi et al.^14^ in advancing our understanding of the mathematical foundations of IFSs. Furthermore, scholars have expanded their investigations to encompass aspects beyond mere aggregation and characteristics. The inventive work of Mahmood and Ali^15^, who presented a novel strategy involving complex q-rung orthopair fuzzy Hamacher aggregation operators, is another source of inspiration for this study. Their use in evaluating cleaner production, especially in gold mines, adds insightful viewpoints to the field of study.

A common theme in IFS generalizations is the connection between the MD (membership degree) ${\mu }_{m}\left(x\right)$(x) and NMD (non membership degree) ${\nu }_{m}\left(x\right)$. A well-known illustration of IFS that has drawn a lot of attention is the IFS interpretation triangle (IFIT), which has been the basis for a number of advancements in Generalized Intuitionistic Fuzzy Sets (GIFS) (as shown in Table 1). Firstly, Mondal and Samanta^16^ introduced GIFS_MS_, which added a condition to the conventional IFS and made it possible to accept instances where ${\mu }_{m}\left(x\right)+{\nu }_{m}\left(x\right)> 1$. However, it is still limited to ${\mu }_{m}\left(x\right)\bigwedge {\nu }_{m}\left(x\right)\le 0.5$. GIFS_L_ was subsequently proposed by Liu^17^, who used linear extensions to interpret the surface. Therefore, it included cases that went beyond ${\mu }_{m}\left(x\right)\bigwedge {\nu }_{m}\left(x\right)>0.5$. with GIFS_MS_ being one such case. Despi et al.^18^ increased the range of possible combinations between ${\mu }_{m}\left(x\right)$ and ${\nu }_{m}\left(x\right)$ by proposing six varieties of GIFS (GIFS1_DOY_ − GIFS6_DOY_). Every GIFS that is suggested offers flexibility when addressing potential situations when ${\mu }_{m}\left(x\right)+{\nu }_{m}\left(x\right)> 1$. Furthermore, GIFS_B_^19^, which is based on the power and root-type of ${\mu }_{m}\left(x\right)$ and ${\nu }_{m}\left(x\right)$, was introduced by Jamkhaneh and Nadarajah. By altering the relationship between ${\mu }_{m}\left(x\right)$ and ${\nu }_{m}\left(x\right)$, the interpretation area under the IFIT is effectively narrowed and broadened. Several well-known IFS extensions that can be found in the body of current literature are also included in GIFS_B_^20–23^. Essentially, these generalizations aim to redefine IFS within the framework of IFIT, thereby increasing the expressive power of ${\mu }_{m}\left(x\right)$ and ${\nu }_{m}\left(x\right)$ degrees. It turns out that the GIFS_B_ concept is a very flexible approach that not only solves the previously mentioned problems but also covers a wide range of specific scenarios of current IFS extensions. This concept can take different forms, but in its formal definition, ${\mu }_{m}\left(x\right)$ and ${\nu }_{m}\left(x\right)$ are parameterized by $\alpha$. When $\alpha$ equals 1, it reverts to the conventional IFS. When α equals 2, it becomes Pythagorean FS (PFS)^24^ or IFS 2-type (IFS2T)^22,25^. It represents Fermatean FS (FFS) when α = 3^26,27^. IFS n-type (IFS-nT) or generalized orthopair FS or q-rung orthopair fuzzy sets are the outcomes of α = n for any positive integer n^23^. Furthermore, it simplifies to the IFS root type (IFSRT) when α is set to 1/2^21^.

**Table 1:** Comparison of some GIFSs (adapted from Pratama et al.^28^ ).

GIFS	Condition	Relation
GIFS_MS_^16^	${\mu }_{m}\left(x\right)\bigwedge {\nu }_{m}\left(x\right)\le 0.5$	$IFS\subset {{\text{GIFS}}}_{{\text{MS}}}$
GIFS_L_^17^	${\mu }_{m}\left(x\right)+{\nu }_{m}\left(x\right)\le 1+\mathrm{L where L}\in [\mathrm{0,1}]$	$IFS\subset {{\text{GIFS}}}_{{\text{MS}}}\subset {{\text{GIFS}}}_{{\text{L}}}$
GIFS1_DOY_^18^	${\mu }_{m}\left(x\right)+{\nu }_{m}\left(x\right)\ge 1$	–
GIFS2_DOY_^18^	${\mu }_{m}\left(x\right)\le {\nu }_{m}\left(x\right)$	–
GIFS3_DOY_^18^	${\mu }_{m}\left(x\right)\ge {\nu }_{m}\left(x\right)$	–
GIFS4_DOY_^18^	$\left\{{\mu }_{m}\left(x\right)+{\nu }_{m}\left(x\right)\ge 1\mathrm{ and }{\mu }_{m}\left(x\right)\ge {\nu }_{m}\left(x\right)\right\}$ or $\left\{{\mu }_{m}\left(x\right)\le {\nu }_{m}\left(x\right) and {\mu }_{m}\left(x\right)+{\nu }_{m}\left(x\right)\le 1\right\}$	–
GIFS5_DOY_^18^	$\left\{{\mu }_{m}\left(x\right)+{\nu }_{m}\left(x\right)\ge 1\mathrm{ and }{\mu }_{m}\left(x\right)\le {\nu }_{m}\left(x\right)\right\}$ or $\left\{{\mu }_{m}\left(x\right)\ge {\nu }_{m}\left(x\right) and {\mu }_{m}\left(x\right)+{\nu }_{m}\left(x\right)\le 1\right\}$	–
GIFS6_DOY_^18^	${\left({\mu }_{m}\left(x\right)\right)}^{2}+{\left({\nu }_{m}\left(x\right)\right)}^{2}\le 1$	–
GIFS_B_^19^	${\left({\mu }_{m}\left(x\right)\right)}^{\alpha }+{\left({\nu }_{m}\left(x\right)\right)}^{\alpha }\le 1$ $where \alpha =n or \frac{1}{n} for n\in {\mathbb{N}}$	If $\alpha =n then IFS\subset {GIFS}_{B}$ If $\alpha =n then IFS\subset {GIFS}_{B}$
GIFS_B_^19,23^	${\left({\mu }_{m}\left(x\right)\right)}^{\alpha }+{\left({\nu }_{m}\left(x\right)\right)}^{\alpha }\le 1$ $where \alpha =n or \frac{1}{n} for n\in {\mathbb{N}}$	If $\alpha =n then q-ROFN\subset {GIFS}_{B}$

This aligns with the innovative work of scholars like Chen and Chang, who extended IFSs to other fields^29^. The heightened flexibility provided by this extension empowers decision-makers to tackle scenarios that require a more comprehensive depiction of uncertainty, akin to the pioneering nature of Atanassov's research on IFSs^2^ and the subsequent advancements in this area^11^. Our primary research objective is to develop and analyze aggregation operations designed specifically for the complex GIFS_B_ model. These operators form the core of our research and have the potential to enhance decision-making capabilities by streamlining the synthesis of information. They aim to improve flexibility, ensuring that the analytical constructs align with individual preferences^30^. Our methodology is informed by previous investigations into aggregation operators developed for interval-valued fuzzy sets^31^. We adeptly adapt and integrate these fundamental principles with the GIFS_B_ structure. The operators discussed in this context demonstrate their practical utility within the dynamic and complex Technology Industry, characterized by continuous evolution and intricacy. Decision-makers in this field face a range of challenges, including resource allocation, market dynamics, and technological advancements. In this setting, ambiguity and subjectivity are prevalent, making the Tech Industry an ideal domain for the application of GIFS_B_s and their associated aggregation operators. Our academic pursuit aligns with the broader domain of decision science, a field in which esteemed scholars like Das, Kar, and Pal have used intuitionistic fuzzy numbers to develop effective decision-making approaches^32^. Our study extends these fundamental principles, applying them to the domain of GIFS_B_s and their innovative aggregation operators. By doing so, we build upon the academic groundwork of researchers exploring similar paths in the realms of fuzzy sets^1^ and generalized fuzzy sets^33^. Farid and Riaz^34^ present q-rung orthopair fuzzy Aczel–Alsina aggregation operators for complex decision scenarios, Riaz and Farid^35^ investigate multicriteria decision-making using spherical fuzzy fairly aggregation operators. In order to improve the efficiency of green supply chains, Riaz and Farid^36^ offer a unique method that emphasizes sustainability by using linear Diophantine fuzzy soft-max aggregation operators. By adding generalized q-rung orthopair fuzzy Einstein interactive geometric aggregation operators, Farid and Riaz^37^ broaden the theoretical underpinnings of decision-making techniques. Taken as a whole, these studies thoroughly investigate the various uses of sophisticated aggregation operators in supply chain management and decision sciences. The primary objective of this study is to carry out a comprehensive investigation into the theoretical foundations of GIFS_B_s and thoroughly examine the structure of our unique aggregation operators: GIFWAA, GIFWGA, GIFOWAA, and GIFOWGA. Finally, we evaluate the practical applicability of these operators. Our aim is to provide a comprehensive framework to help decision-makers efficiently navigate complex decision-making processes by integrating theory and application. This methodology is not limited to the dynamic Tech Industry but extends to other sectors that value data-driven analysis and informed policymaking.

## Preliminaries

In this paper

### Definition 2.1

^2^ An intuitionistic fuzzy set (IFS) M in X is defined as

$M=\left\{\left(x,{\mu }_{M}\left(x\right),{\nu }_{M}\left(x\right)\right)\right|x\in X\}$_._ Where ${\mu }_{M}\left(x\right),{\nu }_{M}\left(x\right)$ are functions of M from X to [0,1] denoted the membership degree and non membership degree respectively, and $0\le {\mu }_{M}\left(x\right)+{\nu }_{M}\left(x\right)\le 1 \forall x\in X$.

### Definition 2.2

^19^ A generalized intuitionistic fuzzy set (GIFS_B_) M in X is defined as

$M=\left\{\left(x,{\mu }_{M}\left(x\right),{\nu }_{M}\left(x\right)\right)\right|x\in X\}$_._ Where ${\mu }_{M}\left(x\right),{\nu }_{M}\left(x\right)$ are functions of M from X to [0,1] denoted the MD and NMD respectively, and $0\le {\left({\mu }_{M}\left(x\right)\right)}^{\alpha }+{\left({\nu }_{M}\left(x\right)\right)}^{\alpha }\le 1 \forall x\in X$, where $\alpha =n or \frac{1}{n}and n\in N and n\ge 0$.

The set $GIFSs(\alpha ,X)$ denotes the collection of all GIFS_B_s. For convenience a generalized intuitionistic fuzzy number (GIFN_B_) is denoted by $m=({\mu }_{m},{\nu }_{m})$.

**Figure 1:**
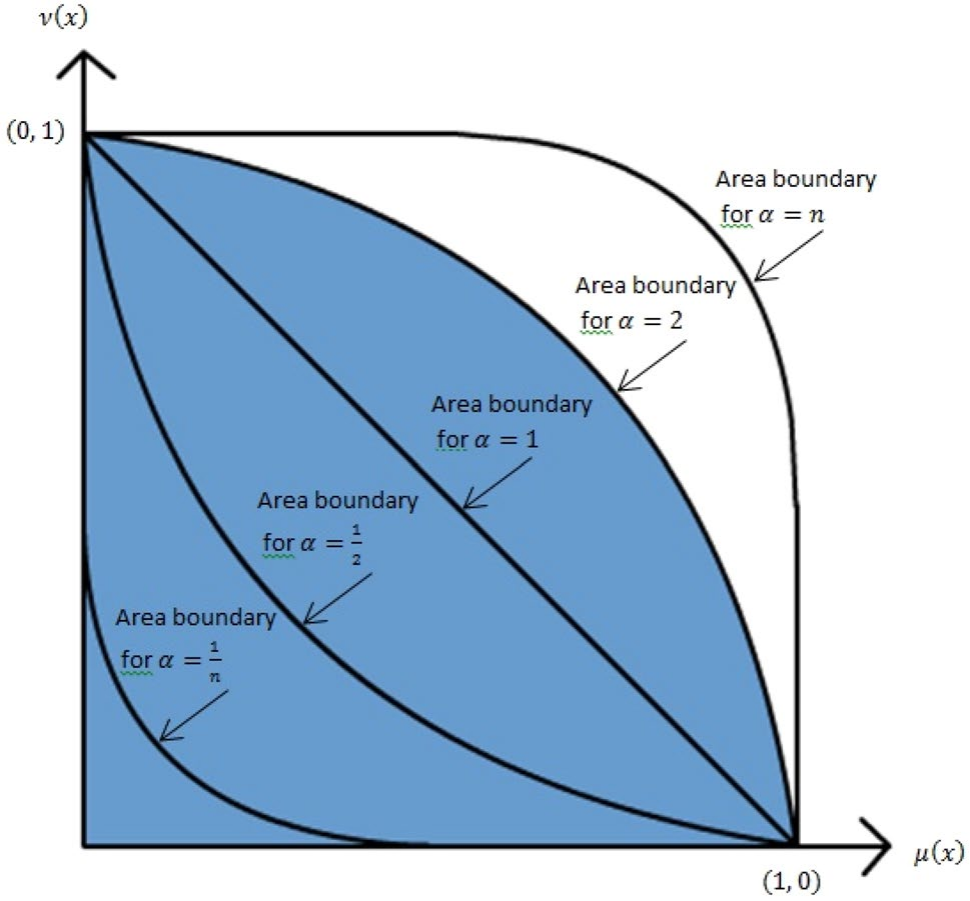
Geometric interpretation of GIFS_B_ for $\alpha =n {\text{or}} \frac{1}{n}$.

Figure 1^28^ illustrates the interpretation area of GIFS_B_ and Table 2 presents its certain special cases related to α.

**Table 2:** Special cases of GIFS_B_.

IFS	Condition	Relation
IFS^2^	*α* = 1	$GIF{S}_{B}B\left(1, X\right)=IFS$
PFS^24^ or IFS of 2nd type^38^	*α* = 2	$IFS\subset GIF{S}_{B}B\left(2, X\right)$
FFS^26^	*α* = 3	$IFS\subset GIF{S}_{B}\left(2, X\right)\subset GIF{S}_{B}\left(3, X\right)$
IFS-nT^39^ or q-ROFS^23^	$\alpha = n,~n \in{\mathbb{N}}$	$IFS\subset GIF{S}_{B}\left(2, X\right)\subset GIF{S}_{B}\left(3, X\right)\subset GIF{S}_{B}\left(n, X\right)$
IFSRT^21^	*α* = 1/2	$GIF{S}_{B}\left(\frac{1}{2}, X\right)\subset IFS\subset IFSRT$

### Definition 2.3

^19^ For an element $x\in X$ The degree of indeterminacy of $x$ to the GIFS_B_s M is defined as

$${\pi }_{M}\left(x\right)={\left(1-{\left({\mu }_{M}\left(x\right)\right)}^{\alpha }+{\left({\nu }_{M}\left(x\right)\right)}^{\alpha }\right)}^{1/\alpha}.$$

### Definition 2.4

^19^ Let M and N be two GIFS_B_s such that $M=\left\{\left(x,{\mu }_{M}\left(x\right),{\nu }_{M}\left(x\right)\right)\right|x\in X\}$ and $N=\left\{\left(x,{\mu }_{N}\left(x\right),{\nu }_{N}\left(x\right)\right)\right|x\in X\}$ **,** then the operations on M and N are defined as,

(i) $$M\subset N$$ iff $${\mu }_{M}\left(x\right){\le \mu }_{N}\left(x\right) and {\nu }_{M}\left(x\right)\ge {\nu }_{N}\left(x\right) \forall x\in X$$_,_
(ii) $$M=N$$ iff $${\mu }_{M}\left(x\right){=\mu }_{N}\left(x\right) and {\nu }_{M}\left(x\right)={\nu }_{N}\left(x\right) \forall x\in X$$_,_
(iii) $$M\cup N=\left\{\left(x,max\left({\mu }_{M}\left(x\right){,\mu }_{N}\left(x\right)\right), min\left({\nu }_{M}\left(x\right),{\nu }_{N}\left(x\right)\right)\right)|x\in X\right\}$$_,_
(iv) $$M\cap N=\left\{\left(x,min\left({\mu }_{M}\left(x\right){,\mu }_{N}\left(x\right)\right), max\left({\nu }_{M}\left(x\right),{\nu }_{N}\left(x\right)\right)\right)|x\in X\right\}$$_,_
(v) $$M \oplus N=\left\{\left(x,{\left\{{\left({\mu }_{M}\left(x\right)\right)}^{\alpha }+{\left({\mu }_{N}\left(x\right)\right)}^{\alpha }-{\left({\mu }_{M}\left(x\right)\right)}^{\alpha }{\left({\mu }_{N}\left(x\right)\right)}^{\alpha }\right\}}^{1/\alpha }, {\nu }_{M}\left(x\right){\nu }_{N}\left(x\right)\right)|x\in X\right\}$$_,_
(vi) $$M \otimes N=\left\{\left(x,{\mu }_{M}\left(x\right){\mu }_{N}\left(x\right), {\left\{{\left({\nu }_{M}\left(x\right)\right)}^{\alpha }+{\left({\nu }_{N}\left(x\right)\right)}^{\alpha }-{\left({\nu }_{M}\left(x\right)\right)}^{\alpha }{\left({\nu }_{N}\left(x\right)\right)}^{\alpha }\right\}}^{1/\alpha}\right)|x\in X\right\}$$_,_
(vii) $$\overline{M }=\left\{\left(x, {\nu }_{M}\left(x\right), {\mu }_{M}\left(x\right)\right)\right|x\in X\}$$_._

## Operations on generalized intuitionistic fuzzy numbers

### Definition 3.1

^19^ Let ${m}_{1}=\left({\mu }_{{m}_{1}},{\nu }_{{m}_{1}}\right), {m}_{2}=\left({\mu }_{{m}_{2}},{\nu }_{{m}_{2}}\right) and m=({\mu }_{m},{\nu }_{m})$ be GIFNs, then the basic operations on GIFNs are defined as follows,

(i) $${m}_{1}\oplus {m}_{2}=\left({\left({\mu }_{{m}_{1}}^{\alpha }+{\mu }_{{m}_{2}}^{\alpha }-{\mu }_{{m}_{1}}^{\alpha }{\mu }_{{m}_{2}}^{\alpha }\right)}^{1/\alpha}, {\nu }_{{m}_{1}}{\nu }_{{m}_{2}}\right)$$,
(ii) $${m}_{1}\otimes {m}_{2}=\left({\mu }_{{m}_{1}}{\mu }_{{m}_{2}}, {\left({\nu }_{{m}_{1}}^{\alpha }+{\nu }_{{m}_{2}}^{\alpha }-{\nu }_{{m}_{1}}^{\alpha }{\nu }_{{m}_{2}}^{\alpha }\right)}^{1/\alpha}\right),$$
(iii) $$\lambda m=\left({\left(1-{\left(1-{\mu }_{m}^{\alpha }\right)}^{\lambda }\right)}^{1/\alpha}, {\nu }_{m}^{\lambda }\right), \lambda >0$$,
(iv) $${m}^{\lambda }=\left({\mu }_{m}^{\lambda }, {\left(1-{\left(1-{\nu }_{m}^{\alpha }\right)}^{\lambda }\right)}^{1/\alpha}\right), \lambda >0$$,
(v) $${m}^{c}=\left({\nu }_{m}, {\mu }_{m}\right)$$.

### Definition 3.2

^33^ Consider the set of GIFNs denoted as ${m}_{i}=\left({\mu }_{{m}_{i}},{\nu }_{{m}_{i}}\right)$, where $i=\mathrm{1,2},\dots n$, then the score function $S({m}_{i})$ and accuracy function $H({m}_{i})$ for GIFNs are defined as,

$$S\left({m}_{i}\right)=\frac{{\left|{\Delta }_{{m}_{i}}\right|}^{1/\alpha}sgn\left({\Delta }_{{m}_{i}}\right)}{2},$$

$$H\left({m}_{i}\right)=\frac{{\left({\left({\mu }_{{m}_{i}}\right)}^{\alpha }+{\left({\nu }_{{m}_{i}}\right)}^{\alpha }\right)}^{1/\alpha}}{2},$$

Where, $${\Delta }_{{m}_{i}}={\left({\mu }_{{m}_{i}}\right)}^{\alpha }-{\left({\nu }_{{m}_{i}}\right)}^{\alpha }$$ and

$$sgn\left({\Delta }_{{m}_{i}}\right)=\left\{\begin{array}{c}-1, {\Delta }_{{m}_{i}}<0,\\ 0, {\Delta }_{{m}_{i}}=0,\\ 1, {\Delta }_{{m}_{i}}>0.\end{array}\right.$$

Obviously, $$S\left({m}_{i}\right)\in [-\mathrm{1,1}]$$ and $$H\left({m}_{i}\right)\in [{0,1}]$$.

(i) If $$S\left({m}_{i}\right)<S\left({m}_{j}\right)$$, then $${m}_{i}<{m}_{j}$$,
(ii) If $S\left({m}_{i}\right)=S\left({m}_{j}\right)$, and
  - $$H\left({m}_{i}\right)<H\left({m}_{j}\right)$$, then $${m}_{i}<{m}_{j}$$,
  - $$H\left({m}_{i}\right)=H\left({m}_{j}\right)$$, then $${m}_{i}={m}_{j}$$.

### Definition 3.3

^40^ The yager class of complements is defined as $c\left(m\right)={(1-{m}^{\alpha })}^{1/\alpha}$, where $\alpha \in (0, \infty )$_._

### Theorem 3.4

*Let* ${m}_{1}=\left({\mu }_{{m}_{1}},{\nu }_{{m}_{1}}\right), {m}_{2}=\left({\mu }_{{m}_{2}},{\nu }_{{m}_{2}}\right) and m=({\mu }_{m},{\nu }_{m})$ *be GIFNs, then for* $\lambda >0$ *the followings are GIFNs*,

(i) $${m}_{1}\oplus {m}_{2}$$,
(ii) $${m}_{1}\otimes {m}_{2}$$,
(iii) $$\lambda m$$, $$\lambda >0$$,
(iv) $${\left(m\right)}^{\lambda }$$, $$\lambda >0$$.

### Proof

(i) Since ${m}_{1}, {m}_{2}$ are GIFNs therefore ${\mu }_{{m}_{1}}, {\mu }_{{m}_{2}},{\nu }_{{m}_{1}},{\nu }_{{m}_{2}}\in [\mathrm{0,1}]$ and $0\le {\mu }_{{m}_{1}}^{\alpha }+{\nu }_{{m}_{1}}^{\alpha }\le 1$ and $0\le {\mu }_{{m}_{2}}^{\alpha }+{\nu }_{{m}_{2}}^{\alpha }\le 1$ ,
  from Definition 3.1
  ${m}_{1}\oplus {m}_{2}=\left({\left({\mu }_{{m}_{1}}^{\alpha }+{\mu }_{{m}_{2}}^{\alpha }-{\mu }_{{m}_{1}}^{\alpha }{\mu }_{{m}_{2}}^{\alpha }\right)}^{1/\alpha}, {\nu }_{{m}_{1}}{\nu }_{{m}_{2}}\right)$, which implies that
  $${\mu }_{{m}_{1}\oplus {m}_{2}}={\left({\mu }_{{m}_{1}}^{\alpha }+{\mu }_{{m}_{2}}^{\alpha }-{\mu }_{{m}_{1}}^{\alpha }{\mu }_{{m}_{2}}^{\alpha }\right)}^{1/\alpha}$$, and $${\nu }_{{m}_{1}\oplus {m}_{2}}={\nu }_{{m}_{1}}{\nu }_{{m}_{2}}$$_._
  Consider,
  $${\mu }_{{m}_{1}\oplus{m}_{2}}^{\alpha }+{\nu }_{{m}_{1}\oplus {m}_{2}}^{\alpha }={\mu }_{{m}_{1}}^{\alpha }+{\mu }_{{m}_{2}}^{\alpha }-{\mu }_{{m}_{1}}^{\alpha }{\mu }_{{m}_{2}}^{\alpha }+{\nu }_{{m}_{1}}^{\alpha }{\nu }_{{m}_{2}}^{\alpha }$$
  $${=\mu }_{{m}_{1}}^{\alpha }(1-{\mu }_{{m}_{2}}^{\alpha })+{\mu }_{{m}_{2}}^{\alpha }+{\nu }_{{m}_{1}}^{\alpha }{\nu }_{{m}_{2}}^{\alpha }\ge 0.$$
  Also,
  $${\mu }_{{m}_{1}\oplus{m}_{2}}^{\alpha }+{\nu }_{{m}_{1}\oplus {m}_{2}}^{\alpha }={\mu }_{{m}_{1}}^{\alpha }+{\mu }_{{m}_{2}}^{\alpha }-{\mu }_{{m}_{1}}^{\alpha }{\mu }_{{m}_{2}}^{\alpha }+{\nu }_{{m}_{1}}^{\alpha }{\nu }_{{m}_{2}}^{\alpha }$$
  $$\le 1-{\nu }_{{m}_{1}}^{\alpha }+1-{\nu }_{{m}_{2}}^{\alpha }-(1-{\nu }_{{m}_{1}}^{\alpha })(1-{\nu }_{{m}_{2}}^{\alpha })+{\nu }_{{m}_{1}}^{\alpha }{\nu }_{{m}_{2}}^{\alpha }$$
  $$=1-{\nu }_{{m}_{1}}^{\alpha }+1-{\nu }_{{m}_{2}}^{\alpha }-1+{\nu }_{{m}_{1}}^{\alpha }+{\nu }_{{m}_{2}}^{\alpha }-{\nu }_{{m}_{1}}^{\alpha }{\nu }_{{m}_{2}}^{\alpha }+{\nu }_{{m}_{1}}^{\alpha }{\nu }_{{m}_{2}}^{\alpha }=1.$$
  Therefore,
$$0\le {\mu }_{{m}_{1}\oplus{m}_{2}}^{\alpha }+{\nu }_{{m}_{1}\oplus{m}_{2}}^{\alpha }\le 1.$$
  Hence ${m}_{1}\oplus {m}_{2}$ is a GIFN.
(ii) It is similar to the proof as in (i) and thus excluded.
(iii) Using Definition 3.1,
  $$\lambda m=\left({\left(1-{\left(1-{\mu }_{m}^{\alpha }\right)}^{\lambda }\right)}^{1/\alpha}, {\nu }_{m}^{\lambda }\right), \lambda >0$$, which implies
  $${\mu }_{\lambda m }={\left(1-{\left(1-{\mu }_{m}^{\alpha }\right)}^{\lambda }\right)}^{1/\alpha}$$, and $${\nu }_{\lambda m}={\nu }_{m}^{\lambda }$$_._
  Consider,
$${\mu }_{\lambda m}^{\alpha }+{\nu }_{\lambda m}^{\alpha }=1-{\left(1-{\mu }_{m}^{\alpha }\right)}^{\lambda }+{\nu }_{m}^{\alpha \lambda }\ge 0,$$
  Also,
$${\mu }_{\lambda m}^{\alpha }+{\nu }_{\lambda m}^{\alpha }=1-{\left(1-{\mu }_{m}^{\alpha }\right)}^{\lambda }+{\nu }_{m}^{\alpha \lambda }$$
$$\le 1-{\left(1-{\mu }_{m}^{\alpha }\right)}^{\lambda }+{\left(1-{\mu }_{m}^{\alpha }\right)}^{\lambda }=1.$$
  Therefore,
$$0\le {\mu }_{\lambda m}^{\alpha }+{\nu }_{\lambda m}^{\alpha }\le 1.$$
  Hence $\lambda m$ is a GIFN.
(iv) It is similar to the proof as in (iii) and thus excluded.

### Theorem 3.5

*Let* $${\lambda }_{1}, {\lambda }_{2}, {\lambda }_{3}>0$$, *then*

(i) $${m}_{1}\oplus{m}_{2}={m}_{2}\oplus {m}_{1}$$,
(ii) $${m}_{1}\otimes{m}_{2}={m}_{2}\otimes {m}_{1}$$,
(iii) $$\lambda \left({m}_{1}\oplus{m}_{2}\right)={\lambda m}_{2}\oplus \lambda {m}_{1}$$,
(iv) $${\left({m}_{1}\otimes{m}_{2}\right)}^{\lambda }={m}_{1}^{\lambda } \otimes {m}_{2}^{\lambda }$$,
(v) $${\lambda }_{1}m \oplus{\lambda }_{2}m={(\lambda }_{1}+ {\lambda }_{2})m$$,
(vi) $${m}^{{\lambda }_{1}} \otimes {m}^{{\lambda }_{2}}={m}^{{(\lambda }_{1}+ {\lambda }_{2})}$$,
(vii) $$\left({m}_{1}\oplus{m}_{2}\right) \oplus m={m}_{1}\oplus\left({m}_{2 }\oplus m\right)$$,
(viii) $${\left({m}^{{\lambda }_{1}}\right)}^{{\lambda }_{2}}={m}^{{\lambda }_{1}{\lambda }_{2}}$$.

### Proof

(i) From Definition 3.1
$${m}_{1}\oplus {m}_{2}=\left({\left({\mu }_{{m}_{1}}^{\alpha }+{\mu }_{{m}_{2}}^{\alpha }-{\mu }_{{m}_{1}}^{\alpha }{\mu }_{{m}_{2}}^{\alpha }\right)}^{1/\alpha}, {\nu }_{{m}_{1}}{\nu }_{{m}_{2}}\right)=\left({\left({\mu }_{{m}_{2}}^{\alpha }+{\mu }_{{m}_{1}}^{\alpha }-{\mu }_{{m}_{2}}^{\alpha }{\mu }_{{m}_{1}}^{\alpha }\right)}^{1/\alpha}, {\nu }_{{m}_{2}}{\nu }_{{m}_{1}}\right)$$
$${m}_{1}\oplus{m}_{2} ={m}_{2}\oplus{m}_{1}.$$
(ii) It is similar to the proof as in (i) and thus excluded.
(iii) From Definition 3.1
$$\lambda \left({m}_{1}\oplus{m}_{2}\right)=\left({\left\{1-{\left(1-\left({\mu }_{{m}_{1}}^{\alpha }+{\mu }_{{m}_{2}}^{\alpha }-{\mu }_{{m}_{1}}^{\alpha }{\mu }_{{m}_{2}}^{\alpha }\right)\right)}^{\lambda }\right\}}^{1/\alpha}, {\nu }_{{m}_{1}}^{\lambda }{\nu }_{{m}_{2}}^{\lambda }\right)$$
$$=\left({\left\{1-{\left(1-{\mu }_{{m}_{1}}^{\alpha }-{\mu }_{{m}_{2}}^{\alpha }+{\mu }_{{m}_{1}}^{\alpha }{\mu }_{{m}_{2}}^{\alpha }\right)}^{\lambda }\right\}}^{1/\alpha}, {\nu }_{{m}_{1}}^{\lambda }{\nu }_{{m}_{2}}^{\lambda }\right)$$
$$=\left({\left\{1-{\left(1-{\mu }_{{m}_{1}}^{\alpha }\right)}^{\lambda }{\left(1-{\mu }_{{m}_{2}}^{\alpha }\right)}^{\lambda }\right\}}^{1/\alpha}, {\nu }_{{m}_{1}}^{\lambda }{\nu }_{{m}_{2}}^{\lambda }\right). $$
  Since, $$\lambda {m}_{1}=\left({\left(1-{\left(1-{\mu }_{{m}_{1}}^{\alpha }\right)}^{\lambda }\right)}^{1/\alpha}, {\nu }_{{m}_{1}}^{\lambda }\right)$$ and
$$\lambda {m}_{2}=\left({\left(1-{\left(1-{\mu }_{{m}_{2}}^{\alpha }\right)}^{\lambda }\right)}^{1/\alpha}, {\nu }_{{m}_{2}}^{\lambda }\right)$$
$${\lambda m}_{2}\oplus \lambda {m}_{1}=\left({\left\{1-{\left(1-{\mu }_{{m}_{1}}^{\alpha }\right)}^{\lambda }+1-{\left(1-{\mu }_{{m}_{2}}^{\alpha }\right)}^{\lambda }-\left(1-{\left(1-{\mu }_{{m}_{1}}^{\alpha }\right)}^{\lambda }\right)\left(1-{\left(1-{\mu }_{{m}_{2}}^{\alpha }\right)}^{\lambda }\right)\right\}}^{1/\alpha}, {\nu }_{{m}_{1}}^{\lambda }{\nu }_{{m}_{2}}^{\lambda }\right)$$
$$=\left({\left\{1-{\left(1-{\mu }_{{m}_{1}}^{\alpha }\right)}^{\lambda }+1-{\left(1-{\mu }_{{m}_{2}}^{\alpha }\right)}^{\lambda }-1+{\left(1-{\mu }_{{m}_{1}}^{\alpha }\right)}^{\lambda }+{\left(1-{\mu }_{{m}_{2}}^{\alpha }\right)}^{\lambda }-{\left(1-{\mu }_{{m}_{1}}^{\alpha }\right)}^{\lambda }{\left(1-{\mu }_{{m}_{2}}^{\alpha }\right)}^{\lambda }\right\}}^{1/\alpha}, {\nu }_{{m}_{1}}^{\lambda }{\nu }_{{m}_{2}}^{\lambda }\right)$$
$$=\left({\left\{1-{\left(1-{\mu }_{{m}_{1}}^{\alpha }\right)}^{\lambda }{\left(1-{\mu }_{{m}_{2}}^{\alpha }\right)}^{\lambda }\right\}}^{1/\alpha}, {\nu }_{{m}_{1}}^{\lambda }{\nu }_{{m}_{2}}^{\lambda }\right). $$
  Hence,
$$\lambda \left({m}_{1}\oplus{m}_{2}\right)={\lambda m}_{2}\oplus\lambda {m}_{1}. $$
(iv) It is similar to the proof as in (iii) and thus excluded.
(v) From Definition 3.1
$${\lambda }_{1}m \oplus{\lambda }_{2}m=\left({\left\{1-{\left(1-{\mu }_{m}^{\alpha }\right)}^{{\lambda }_{1}}+1-{\left(1-{\mu }_{m}^{\alpha }\right)}^{{\lambda }_{2}}-\left(1-{\left(1-{\mu }_{m}^{\alpha }\right)}^{{\lambda }_{1}}\right)\left(1-{\left(1-{\mu }_{m}^{\alpha }\right)}^{{\lambda }_{2}}\right)\right\}}^{1/\alpha}, {\nu }_{m}^{{\lambda }_{1}}{\nu }_{m}^{{\lambda }_{2}}\right)$$
$$=\left({\left\{1-{\left(1-{\mu }_{m}^{\alpha }\right)}^{{\lambda }_{1}}+1-{\left(1-{\mu }_{m}^{\alpha }\right)}^{{\lambda }_{2}}-1+{\left(1-{\mu }_{m}^{\alpha }\right)}^{{\lambda }_{1}}+{\left(1-{\mu }_{m}^{\alpha }\right)}^{{\lambda }_{2}}-{\left(1-{\mu }_{m}^{\alpha }\right)}^{{\lambda }_{1}}{\left(1-{\mu }_{m}^{\alpha }\right)}^{{\lambda }_{2}}\right\}}^{1/\alpha}, {\nu }_{m}^{{\lambda }_{1}}{\nu }_{m}^{{\lambda }_{2}}\right)$$
$$=\left({\left\{1-{\left(1-{\mu }_{m}^{\alpha }\right)}^{{\lambda }_{1}+{\lambda }_{2}}\right\}}^{1/\alpha}, {\nu }_{m}^{{\lambda }_{1}+{\lambda }_{2}}\right)$$
$$={(\lambda }_{1}+ {\lambda }_{2})m. $$
  Hence,
$${\lambda }_{1}m \oplus{\lambda }_{2}m={(\lambda }_{1}+ {\lambda }_{2})m.$$
(vi) It is similar to the proof as in (v) and thus excluded.
(vii) From Definition 3.1
$$\left({m}_{1}\oplus{m}_{2}\right) \oplus m=\left({\left\{{\mu }_{{m}_{1}}^{\alpha }+{\mu }_{{m}_{2}}^{\alpha }-{\mu }_{{m}_{1}}^{\alpha }{\mu }_{{m}_{2}}^{\alpha }+{\mu }_{m}^{\alpha }-\left({\mu }_{{m}_{1}}^{\alpha }+{\mu }_{{m}_{2}}^{\alpha }-{\mu }_{{m}_{1}}^{\alpha }{\mu }_{{m}_{2}}^{\alpha }\right){\mu }_{m}^{\alpha }\right\}}^{1/\alpha}, \left({\nu }_{{m}_{1}}{\nu }_{{m}_{2}}\right){\nu }_{m}\right)$$
$$=\left({\left\{{\mu }_{{m}_{1}}^{\alpha }+{\mu }_{{m}_{2}}^{\alpha }-{\mu }_{{m}_{1}}^{\alpha }{\mu }_{{m}_{2}}^{\alpha }+{\mu }_{m}^{\alpha }-{\mu }_{{m}_{1}}^{\alpha }{\mu }_{m}^{\alpha }-{\mu }_{{m}_{2}}^{\alpha }{\mu }_{m}^{\alpha }+{\mu }_{{m}_{1}}^{\alpha }{\mu }_{{m}_{2}}^{\alpha }{\mu }_{m}^{\alpha }\right\}}^{1/\alpha}, {\nu }_{{m}_{1}}{\nu }_{{m}_{2}}{\nu }_{m}\right)$$
$$=\left({\left\{{\mu }_{{m}_{1}}^{\alpha }+{\mu }_{{m}_{2}}^{\alpha }+{\mu }_{m}^{\alpha }-{\mu }_{{m}_{2}}^{\alpha }{\mu }_{m}^{\alpha }-{\mu }_{{m}_{1}}^{\alpha }{\mu }_{{m}_{2}}^{\alpha }-{\mu }_{{m}_{1}}^{\alpha }{\mu }_{m}^{\alpha }+{\mu }_{{m}_{1}}^{\alpha }{\mu }_{{m}_{2}}^{\alpha }{\mu }_{m}^{\alpha }\right\}}^{1/\alpha}, {\nu }_{{m}_{1}}{\nu }_{{m}_{2}}{\nu }_{m}\right)$$
$$=\left({\left\{{\mu }_{{m}_{1}}^{\alpha }+{\mu }_{{m}_{2}}^{\alpha }+{\mu }_{m}^{\alpha }-{\mu }_{{m}_{2}}^{\alpha }{\mu }_{m}^{\alpha }-{\mu }_{{m}_{1}}^{\alpha }\left({\mu }_{{m}_{2}}^{\alpha }+{\mu }_{m}^{\alpha }-{\mu }_{{m}_{2}}^{\alpha }{\mu }_{m}^{\alpha }\right)\right\}}^{1/\alpha}, {\nu }_{{m}_{1}}\left({\nu }_{{m}_{2}}{\nu }_{m}\right)\right)$$
$$={m}_{1}\oplus\left({m}_{2 }\oplus m\right).$$
  Hence,
$$\left({m}_{1}\oplus{m}_{2}\right) \oplus m={m}_{1}\oplus\left({m}_{2 }\oplus m\right).$$
(vii) From Definition 3.1
$${m}^{{\lambda }_{1}}=\left({\mu }_{m}^{{\lambda }_{1}}, {\left(1-{\left(1-{\nu }_{m}^{\alpha }\right)}^{{\lambda }_{1}}\right)}^{1/\alpha}\right)$$
$${\left({m}^{{\lambda }_{1}}\right)}^{{\lambda }_{2}}=\left({\left({\mu }_{m}^{{\lambda }_{1}}\right)}^{{\lambda }_{2}}, {\left\{1-{\left(1-\left(1-{\left(1-{\nu }_{m}^{\alpha }\right)}^{{\lambda }_{1}}\right)\right)}^{{\lambda }_{2}}\right\}}^{1/\alpha}\right)$$
$$=\left({\mu }_{m}^{{\lambda }_{1}{\lambda }_{2}}, {\left\{1-{\left(1-1+{\left(1-{\nu }_{m}^{\alpha }\right)}^{{\lambda }_{1}}\right)}^{{\lambda }_{2}}\right\}}^{1/\alpha}\right)$$
$$=\left({\mu }_{m}^{{\lambda }_{1}{\lambda }_{2}}, {\left\{1-{\left(1-{\nu }_{m}^{\alpha }\right)}^{{\lambda }_{1}{\lambda }_{2}}\right\}}^{1/\alpha}\right)$$
$$={m}^{{\lambda }_{1}{\lambda }_{2}}. $$
  Hence,
$${\left({m}^{{\lambda }_{1}}\right)}^{{\lambda }_{2}}={m}^{{\lambda }_{1}{\lambda }_{2}}.$$

## Weighted averaging aggregation operators with GIFNs

In this section , we present two aggregation operators, GIFWAA operator and GIFWGA operator. These operators are formulated in accordance with the operational principles governing GIFNs. We also delve into an exploration of key characteristics of these operators, providing a comprehensive understanding of their mathematical foundations within our research framework.

### Definition 4.1

Consider the set of GIFNs denoted as ${m}_{i}=\left({\mu }_{{m}_{i}},{\nu }_{{m}_{i}}\right)$, where $i=\mathrm{1,2},3\dots n$, then the generalized intuitionistic fuzzy weighted averaging aggregation (GIFWAA) operator is a mapping $GIFWAA: {m}^{n}\to m$ defined as

$$GIFWA{A}_{w}\left({m}_{1},{m}_{2},\dots {m}_{n}\right)={\oplus }_{i=1}^{n}{w}_{i}{m}_{i}$$

where $w={({w}_{1},{w}_{2}\dots ,{w}_{n})}^{T}$ is the weight vector of ${m}_{i}$, with ${w}_{i}\in \left[\mathrm{0,1}\right] and \sum_{i=1}^{n}{w}_{i}=1.$ In particular case, when $w={\left(\frac{1}{n}, \frac{1}{n},\dots , \frac{1}{n}\right)}^{T}$, then GIFWAA operator reduces to generalized intuitionistic fuzzy averaging aggregation (GIFAA) operator.

$$GIFAA\left({m}_{1},{m}_{2},\dots {m}_{n}\right)=\frac{1}{n}{\oplus }_{i=1}^{n}{m}_{i}.$$

### Theorem 4.2

*Consider the set of GIFNs denoted as* ${m}_{i}=\left({\mu }_{{m}_{i}},{\nu }_{{m}_{i}}\right)$, *where* $i=\mathrm{1,2},\dots n$ *with the corresponding weight vector* $w={({w}_{1},{w}_{2}\dots ,{w}_{n})}^{T}$ *of* ${m}_{i}$, *such that* ${w}_{i}\in \left[\mathrm{0,1}\right], and \sum_{i=1}^{n}{w}_{i}=1$, *then the aggregated value of* ${m}_{i}$ *by using GIFWAA operator is also a GIFN, where*

$$GIFWA{A}_{w}\left({m}_{1},{m}_{2},\dots {m}_{n}\right)=\left({\left\{1-{\prod }_{i=1}^{n}{\left(1-{\mu }_{{m}_{i}}^{\alpha }\right)}^{{w}_{i}}\right\}}^{1/\alpha}, {\prod }_{i=1}^{n}{\nu }_{{m}_{i}}^{{w}_{i}}\right)$$

where $\alpha =n or \frac{1}{n} and n\in {\mathbb{N}},n>0$. In particular if ${\mu }_{{m}_{i}}^{\alpha }=1-{\nu }_{{m}_{i}}^{\alpha }$, then,

$$GIFWA{A}_{w}\left({m}_{1},{m}_{2},\dots {m}_{n}\right)=\left({\left\{1-{\prod }_{i=1}^{n}{\left(1-{\mu }_{{m}_{i}}^{\alpha }\right)}^{{w}_{i}}\right\}}^{1/\alpha}, {\left\{{\prod }_{i=1}^{n}{\left(1-{\mu }_{{m}_{i}}^{\alpha }\right)}^{{w}_{i}}\right\}}^{1/\alpha}\right).$$

### Proof

We prove by using Inductive reasoning,

Case 1: when $$n=2$$

$$GIFWA{A}_{w}\left({m}_{1},{m}_{2}\right)={w}_{1}{m}_{1}\oplus {{w}_{2}m}_{2}$$

From Theorem 3.5, it is clear that ${w}_{1}{m}_{1}, {{w}_{2}m}_{2}\in GIFNs$, also that ${w}_{1}{m}_{1}\oplus {{w}_{2}m}_{2}\in GIFNs$. From Definition 3.1, we get

$${w}_{1}{m}_{1}=\left({\left(1-{\left(1-{\mu }_{{m}_{1}}^{\alpha }\right)}^{{w}_{1}}\right)}^{1/\alpha}, {\nu }_{{m}_{1}}^{{w}_{1}}\right),$$

$${w}_{2}{m}_{2}=\left({\left(1-{\left(1-{\mu }_{{m}_{2}}^{\alpha }\right)}^{{w}_{2}}\right)}^{1/\alpha}, {\nu }_{{m}_{2}}^{{w}_{2}}\right)$$, then

$$GIFWA{A}_{w}\left({m}_{1},{m}_{2}\right)=\left({\left\{1-{\left(1-{\mu }_{{m}_{1}}^{\alpha }\right)}^{{w}_{1}}+1-{\left(1-{\mu }_{{m}_{2}}^{\alpha }\right)}^{{w}_{2}}-\left(1-{\left(1-{\mu }_{{m}_{1}}^{\alpha }\right)}^{{w}_{1}}\right)\left(1-{\left(1-{\mu }_{{m}_{2}}^{\alpha }\right)}^{{w}_{2}}\right)\right\}}^{1/\alpha}, {\nu }_{{m}_{1}}^{{w}_{1}}{\nu }_{{m}_{2}}^{{w}_{2}}\right)$$

$$=\left({\left\{1-{\left(1-{\mu }_{{m}_{1}}^{\alpha }\right)}^{{w}_{1}}+1-{\left(1-{\mu }_{{m}_{2}}^{\alpha }\right)}^{{w}_{2}}-1+{\left(1-{\mu }_{{m}_{1}}^{\alpha }\right)}^{{w}_{1}}+{\left(1-{\mu }_{{m}_{2}}^{\alpha }\right)}^{{w}_{2}}-{\left(1-{\mu }_{{m}_{1}}^{\alpha }\right)}^{{w}_{1}}{\left(1-{\mu }_{{m}_{2}}^{\alpha }\right)}^{{w}_{2}}\right\}}^{1/\alpha}, {\nu }_{{m}_{1}}^{{w}_{1}}{\nu }_{{m}_{2}}^{{w}_{2}}\right)$$

$$=\left({\left\{1-{\left(1-{\mu }_{{m}_{1}}^{\alpha }\right)}^{{w}_{1}}{\left(1-{\mu }_{{m}_{2}}^{\alpha }\right)}^{{w}_{2}}\right\}}^{1/\alpha}, {\nu }_{{m}_{1}}^{{w}_{1}}{\nu }_{{m}_{2}}^{{w}_{2}}\right)$$

$$=\left({\left\{1-{\prod }_{i=1}^{2}{\left(1-{\mu }_{{m}_{i}}^{\alpha }\right)}^{{w}_{i}}\right\}}^{1/\alpha}, {\prod }_{i=1}^{2}{\nu }_{{m}_{i}}^{{w}_{i}}\right),$$

Case 2: Suppose, Theorem 4.2 is true for $n=k$, that is

$$GIFWA{A}_{w}\left({m}_{1},{m}_{2},\dots {m}_{k}\right)=\left({\left\{1-{\prod }_{i=1}^{k}{\left(1-{\mu }_{{m}_{i}}^{\alpha }\right)}^{{w}_{i}}\right\}}^{1/\alpha}, {\prod }_{i=1}^{k}{\nu }_{{m}_{i}}^{{w}_{i}}\right)$$, and also $$GIFWA{A}_{w}\left({m}_{1},{m}_{2},\dots {m}_{k}\right)\in GIFNs$$.

Consider,

$$GIFWA{A}_{w}\left({m}_{1},{m}_{2},\dots {m}_{k}, {m}_{k+1}\right)={w}_{1}{m}_{1}\oplus {{w}_{2}m}_{2}\dots \oplus{{w}_{k}m}_{k} \oplus {{w}_{k+1}m}_{k+1}$$

$$=\left({\left\{1-{\prod }_{i=1}^{k}{\left(1-{\mu }_{{m}_{i}}^{\alpha }\right)}^{{w}_{i}}\right\}}^{1/\alpha}, {\prod }_{i=1}^{k}{\nu }_{{m}_{i}}^{{w}_{i}}\right)\oplus \left({\left\{1-{\left(1-{\mu }_{{m}_{k+1}}^{\alpha }\right)}^{{w}_{k+1}}\right\}}^{1/\alpha}, {\nu }_{{m}_{k+1}}^{{w}_{k+1}}\right)$$

$$=\left(1-{\prod }_{i=1}^{k}{\left(1-{\mu }_{{m}_{i}}^{\alpha }\right)}^{{w}_{i}}+1-{\left(1-{\mu }_{{m}_{k+1}}^{\alpha }\right)}^{{w}_{k+1}}-\left(1-{\prod }_{i=1}^{k}{\left(1-{\mu }_{{m}_{i}}^{\alpha }\right)}^{{w}_{i}}\right)\left(1-{\left(1-{\mu }_{{m}_{k+1}}^{\alpha }\right)}^{{w}_{k+1}}\right), {\prod }_{i=1}^{k}{\nu }_{{m}_{i}}^{{w}_{i}}{\nu }_{{m}_{k+1}}^{{w}_{k+1}}\right)$$

$$=\left(1-{\prod }_{i=1}^{k}{\left(1-{\mu }_{{m}_{i}}^{\alpha }\right)}^{{w}_{i}}+1-{\left(1-{\mu }_{{m}_{k+1}}^{\alpha }\right)}^{{w}_{k+1}}-1+{\prod }_{i=1}^{k}{\left(1-{\mu }_{{m}_{i}}^{\alpha }\right)}^{{w}_{i}}+{\left(1-{\mu }_{{m}_{k+1}}^{\alpha }\right)}^{{w}_{k+1}}-{\prod }_{i=1}^{k}{\left(1-{\mu }_{{m}_{i}}^{\alpha }\right)}^{{w}_{i}}{\left(1-{\mu }_{{m}_{k+1}}^{\alpha }\right)}^{{w}_{k+1}}, {\prod }_{i=1}^{k+1}{\nu }_{{m}_{i}}^{{w}_{i}}\right)$$

$$=\left(1-{\prod }_{i=1}^{k+1}{\left(1-{\mu }_{{m}_{i}}^{\alpha }\right)}^{{w}_{i}}, {\prod }_{i=1}^{k+1}{\nu }_{{m}_{i}}^{{w}_{i}}\right).$$

Which is also a GIFN. Therefore, Theorem 4.2 is true for *n* = *k* + 1.

Hence, Theorem 4.2 is true for all $n$.

### Definition 4.3

Consider the set of GIFNs denoted as ${m}_{i}=\left({\mu }_{{m}_{i}},{\nu }_{{m}_{i}}\right)$, where $i=\mathrm{1,2},\dots n$, then the generalized intuitionistic fuzzy weighted geometric aggregation (GIFWGA) operator is a mapping $GIFWGA: {m}^{n}\to m$ defined as

$$GIFWG{A}_{w}\left({m}_{1},{m}_{2},\dots {m}_{n}\right)={\otimes}_{i=1}^{n}{\left({m}_{i}\right)}^{{w}_{i}}$$

where $w={({w}_{1},{w}_{2}\dots ,{w}_{n})}^{T}$ is the weight vector of ${m}_{i}$, with ${w}_{i}\in \left[\mathrm{0,1}\right] and \sum_{i=1}^{n}{w}_{i}=1.$ In particular case, when $w={\left(\frac{1}{n}, \frac{1}{n},\dots , \frac{1}{n}\right)}^{T}$, then GIFWGA operator reduces to generalized intuitionistic fuzzy geometric aggregation (GIFGA) operator.

$$GIFGA\left({m}_{1},{m}_{2},\dots {m}_{n}\right)={\left({\otimes}_{i=1}^{n}{m}_{i}\right)}^{1/n}$$

### Theorem 4.4

*Consider the set of GIFNs denoted as* ${m}_{i}=\left({\mu }_{{m}_{i}},{\nu }_{{m}_{i}}\right)$, *where* $i=\mathrm{1,2},\dots n$ *with the corresponding exponential weight vector* $w={({w}_{1},{w}_{2}\dots ,{w}_{n})}^{T}$ *of* ${m}_{i}$, *such that* ${w}_{i}\in \left[\mathrm{0,1}\right] , and \sum_{i=1}^{n}{w}_{i}=1$, *then the aggregated value of* ${m}_{i}$ *by using GIFWGA operator is also a GIFN, where*

$$GIFWG{A}_{w}\left({m}_{1},{m}_{2},\dots {m}_{n}\right)=\left({\prod }_{i=1}^{n}{\mu }_{{m}_{i}}^{{w}_{i}}, {\left\{1-{\prod }_{i=1}^{n}{\left(1-{\nu }_{{m}_{i}}^{\alpha }\right)}^{{w}_{i}}\right\}}^{1/\alpha}\right)$$

where $\alpha =n or \frac{1}{n} and n\in {\mathbb{N}},n>0$. In particular if ${\mu }_{{m}_{i}}^{\alpha }=1-{\nu }_{{m}_{i}}^{\alpha }$. Then,

$$GIFWG{A}_{w}\left({m}_{1},{m}_{2},\dots {m}_{n}\right)=\left({\prod }_{i=1}^{n}{\mu }_{{m}_{i}}^{{w}_{i}}, {\left\{1-{\prod }_{i=1}^{n}{\mu }_{{m}_{i}}^{\alpha {w}_{i}}\right\}}^{1/\alpha}\right).$$

### Proof

We prove Theorem 4.4 by using Inductive reasoning,

Case 1: when $$n=2$$

$$GIFWG{A}_{w}\left({m}_{1},{m}_{2}\right)={\left({m}_{1}\right)}^{{w}_{1}} \otimes {\left({m}_{2}\right)}^{{w}_{2}}$$

From Theorem 3.5, it is clear that ${\left({m}_{1}\right)}^{{w}_{1}}, {\left({m}_{2}\right)}^{{w}_{2}}\in GIFNs$, also that ${\left({m}_{1}\right)}^{{w}_{1}} \otimes {\left({m}_{2}\right)}^{{w}_{2}}\in GIFNs$. From Definition 3.1, we get

$${{m}_{1}}^{{w}_{1}}=\left( {\mu }_{{m}_{1}}^{{w}_{1}}, {\left(1-{\left(1-{\nu }_{{m}_{1}}^{\alpha }\right)}^{{w}_{1}}\right)}^{1/\alpha}\right){{m}_{2}}^{{w}_{2}}=\left({\mu }_{{m}_{2}}^{{w}_{2}}, {\left(1-{\left(1-{\nu }_{{m}_{2}}^{\alpha }\right)}^{{w}_{2}}\right)}^{1/\alpha}\right),$$

Then,

$$GIFWG{A}_{w}\left({m}_{1},{m}_{2}\right)=\left({\mu }_{{m}_{1}}^{{w}_{1}}{{\mu }_{{m}_{2}}^{{w}_{2}}, \left\{1-{\left(1-{\nu }_{{m}_{1}}^{\alpha }\right)}^{{w}_{1}}+1-{\left(1-{\nu }_{{m}_{2}}^{\alpha }\right)}^{{w}_{2}}-\left(1-{\left(1-{\nu }_{{m}_{1}}^{\alpha }\right)}^{{w}_{1}}\right)\left(1-{\left(1-{\nu }_{{m}_{2}}^{\alpha }\right)}^{{w}_{2}}\right)\right\}}^{1/\alpha}\right)$$

$$=\left({\mu }_{{m}_{1}}^{{w}_{1}}{{\mu }_{{m}_{2} }^{{w}_{2}} , \left\{1-{\left(1-{\nu }_{{m}_{1}}^{\alpha }\right)}^{{w}_{1}}+1-{\left(1-{\nu }_{{m}_{2}}^{\alpha }\right)}^{{w}_{2}}-1+{\left(1-{\nu }_{{m}_{1}}^{\alpha }\right)}^{{w}_{1}}+{\left(1-{\nu }_{{m}_{2}}^{\alpha }\right)}^{{w}_{2}}-{\left(1-{\nu }_{{m}_{1}}^{\alpha }\right)}^{{w}_{1}}{\left(1-{\nu }_{{m}_{2}}^{\alpha }\right)}^{{w}_{2}}\right\}}^{1/\alpha}\right)$$

$$=\left({\mu }_{{m}_{1}}^{{w}_{1}}{{\mu }_{{m}_{2} }^{{w}_{2}} , \left\{1-{\left(1-{\nu }_{{m}_{1}}^{\alpha }\right)}^{{w}_{1}}{\left(1-{\nu }_{{m}_{2}}^{\alpha }\right)}^{{w}_{2}}\right\}}^{1/\alpha}\right)$$

$$=\left({\mu }_{{m}_{1}}^{{w}_{1}}{{\mu }_{{m}_{2} }^{{w}_{2}} , \left\{1-{\left(1-{\nu }_{{m}_{1}}^{\alpha }\right)}^{{w}_{1}}{\left(1-{\nu }_{{m}_{2}}^{\alpha }\right)}^{{w}_{2}}\right\}}^{1/\alpha}\right)$$

$$=\left({\prod }_{i=1}^{2}{\mu }_{{m}_{i}}^{{w}_{i}}, {\left\{1-{\prod }_{i=1}^{2}{\left(1-{\nu }_{{m}_{i}}^{\alpha }\right)}^{{w}_{i}}\right\}}^{1/\alpha}\right)$$

Case 2: Suppose, theorem 4.4 is true for $n=k$, that is

$$GIFWG{A}_{w}\left({m}_{1},{m}_{2},\dots {m}_{k}\right)=\left({\prod }_{i=1}^{k}{\mu }_{{m}_{i}}^{{w}_{i}}, {\left\{1-{\prod }_{i=1}^{k}{\left(1-{\nu }_{{m}_{i}}^{\alpha }\right)}^{{w}_{i}}\right\}}^{1/\alpha}\right)$$, and also $$GIFWG{A}_{w}\left({m}_{1},{m}_{2},\dots {m}_{k}\right)\in GIFNs$$.

Consider,

$$GIFWG{A}_{w}\left({m}_{1},{m}_{2},\dots {m}_{k}, {m}_{k+1}\right)={{m}_{1}}^{{w}_{1}} \otimes {{m}_{2}}^{{w}_{2}}\otimes ,\dots \otimes {{m}_{k}}^{{w}_{k}} \oplus {{m}_{k+1}}^{{w}_{k+1}}$$

$$=\left({\prod }_{i=1}^{k}{\mu }_{{m}_{i}}^{{w}_{i}}, {\left\{1-{\prod }_{i=1}^{k}{\left(1-{\nu }_{{m}_{i}}^{\alpha }\right)}^{{w}_{i}}\right\}}^{1/\alpha} \right)\oplus \left({\mu }_{{m}_{k+1}}^{{w}_{k+1}}, {\left\{1-{\left(1-{\nu }_{{m}_{k+1}}^{\alpha }\right)}^{{w}_{k+1}}\right\}}^{1/\alpha}\right)$$

$$ \begin{aligned}&=\left({\prod }_{i=1}^{k}{\mu }_{{m}_{i}}^{{w}_{i}}{\mu }_{{m}_{k+1}}^{{w}_{k+1}}, {\left\{1-{\prod }_{i=1}^{k}{\left(1-{\nu }_{{m}_{i}}^{\alpha }\right)}^{{w}_{i}}+1-{\left(1-{\nu }_{{m}_{k+1}}^{\alpha }\right)}^{{w}_{k+1}}\right.}\right. \\  & \quad\left.{\left.-\left(1-{\prod }_{i=1}^{k}{\left(1-{\nu }_{{m}_{i}}^{\alpha }\right)}^{{w}_{i}}\right)\left(1-{\left(1-{\nu }_{{m}_{k+1}}^{\alpha }\right)}^{{w}_{k+1}}\right)\right\}}^{1/\alpha}\right)\end{aligned} $$

$$ \begin{aligned}&=\left({\prod }_{i=1}^{k+1}{\mu }_{{m}_{i}}^{{w}_{i}},{\left\{1-{\prod }_{i=1}^{k}{\left(1-{\nu }_{{m}_{i}}^{\alpha }\right)}^{{w}_{i}}+1-{\left(1-{\nu }_{{m}_{k+1}}^{\alpha }\right)}^{{w}_{k+1}}-1+{\prod }_{i=1}^{k}{\left(1-{\nu }_{{m}_{i}}^{\alpha }\right)}^{{w}_{i}}\right. }\right. \\ & \quad\left.{\left. +{\left(1-{\nu }_{{m}_{k+1}}^{\alpha }\right)}^{{w}_{k+1}}-{\prod }_{i=1}^{k}{\left(1-{\nu }_{{m}_{i}}^{\alpha }\right)}^{{w}_{i}}{\left(1-{\nu }_{{m}_{k+1}}^{\alpha }\right)}^{{w}_{k+1}}\right\}}^{1/\alpha}\right)\end{aligned} $$

$$=\left({\prod }_{i=1}^{k+1}{\mu }_{{m}_{i}}^{{w}_{i}},{\left\{1-{\prod }_{i=1}^{k+1}{\left(1-{\nu }_{{m}_{i}}^{\alpha }\right)}^{{w}_{i}}\right\}}^{1/\alpha}\right).$$

Which is also a GIFN. Therefore, Theorem 4.4 is true for *n* = *k* + 1. 

Hence Theorem 4.4 is true for all $n$.

### Example 4.5

Certainly, let's consider a business scenario. Picture a team consisting of five market analysts, labeled as ${m}_{1}, {m}_{2}, {m}_{3}, {m}_{4}$ and ${m}_{5}$, each specializing in a distinct market segment. These analysts provide their insights into the potential success of a product P using generalized intuitionistic fuzzy information: ${m}_{1}=\left(0.5, 0.7\right), {m}_{2}=\left(0.4, 0.8\right), {m}_{3}=\left(0.9, 0.2\right), {m}_{4}=\left(0.7, 0.4\right)$ and ${m}_{5}=\left(0.6, 0.5\right)$. To reflect the varying importance of each analyst's expertise, a corresponding weight vector is assigned as w = (0.3, 0.2, 0.1, 0.2, 0.2). By employing the GIFWAA operator, a holistic evaluation of the potential success of the product P is generated. This synthesized analysis aids business leaders in making informed decisions regarding resource allocation and strategic direction for maximizing overall profitability and market share.

For $$\alpha =2$$

$$GIFWA{A}_{w}\left({m}_{1},{m}_{2}, {m}_{3}, {m}_{4}, {m}_{5}\right)={w}_{1}{m}_{1}\oplus {{w}_{2}m}_{2}\oplus {{w}_{3}m}_{3}\oplus {{w}_{4}m}_{4}\oplus {{w}_{5}m}_{5}$$

$$ \begin{aligned}& =\left({\left\{1-{\left(1-{\left(0.5\right)}^{2}\right)}^{0.3}{\left(1-{\left(0.4\right)}^{2}\right)}^{0.2}{\left(1-{\left(0.9\right)}^{2}\right)}^{0.1}\right.}\right. \\ & \quad  \left.{\left.{\left(1-{\left(0.7\right)}^{2}\right)}^{0.2}{\left(1-{\left(0.6\right)}^{2}\right)}^{0.2}\right\}}^{1/2}, {\left(0.7\right)}^{0.3}{\left(0.8\right)}^{0.2}{\left(0.2\right)}^{0.1}{\left(0.4\right)}^{0.2}{\left(0.5\right)}^{0.2}\right)\end{aligned} $$

$$=\left(0.63262, 0.53022\right)$$

$$GIFWG{A}_{w}\left({m}_{1},{m}_{2}, {m}_{3}, {m}_{4}, {m}_{5}\right)={{m}_{1}}^{{w}_{1}}\otimes {{m}_{2}}^{{w}_{2}}\otimes {{m}_{3}}^{{w}_{3}}\otimes {{m}_{4}}^{{w}_{4}}\otimes {{m}_{5}}^{{w}_{5}}$$

$$ \begin{aligned}&=\left({\left(0.5\right)}^{0.3}{\left(0.4\right)}^{0.2}{\left(0.9\right)}^{0.1}{\left(0.7\right)}^{0.2}{\left(0.6\right)}^{0.2}, {\left\{1-{\left(1-{\left(0.7\right)}^{2}\right)}^{0.3}\right.}\right.\\ & \quad \left.{\left.{\left(1-{\left(0.8\right)}^{2}\right)}^{0.2}{\left(1-{\left(0.2\right)}^{2}\right)}^{0.1}{\left(1-{\left(0.4\right)}^{2}\right)}^{0.2}{\left(1-{\left(0.5\right)}^{2}\right)}^{0.2}\right\}}^{1/2}\right)\end{aligned} $$

$$=(0.56257, 0.62863)$$

For $$\alpha =3$$

$$GIFWA{A}_{w}\left({m}_{1},{m}_{2}, {m}_{3}, {m}_{4}, {m}_{5}\right)={w}_{1}{m}_{1}\oplus {{w}_{2}m}_{2}\oplus {{w}_{3}m}_{3}\oplus {{w}_{4}m}_{4}\oplus {{w}_{5}m}_{5}$$

$$ \begin{aligned} & =\left({\left\{1-{\left(1-{\left(0.5\right)}^{3}\right)}^{0.3}{\left(1-{\left(0.4\right)}^{3}\right)}^{0.2}{\left(1-{\left(0.9\right)}^{3}\right)}^{0.1}\right.}\right.\\ & \quad \left.{\left.{\left(1-{\left(0.7\right)}^{3}\right)}^{0.2}{\left(1-{\left(0.6\right)}^{3}\right)}^{0.2}\right\}}^{1/3}, {\left(0.7\right)}^{0.3}{\left(0.8\right)}^{0.2}{\left(0.2\right)}^{0.1}{\left(0.4\right)}^{0.2}{\left(0.5\right)}^{0.1}\right)\end{aligned} $$

$$=\left(0.64739, 0.53022\right)$$

$$GIFWG{A}_{w}\left({m}_{1},{m}_{2}, {m}_{3}, {m}_{4}, {m}_{5}\right)={{m}_{1}}^{{w}_{1}}\otimes {{m}_{2}}^{{w}_{2}}\otimes {{m}_{3}}^{{w}_{3}}\otimes {{m}_{4}}^{{w}_{4}}\otimes {{m}_{5}}^{{w}_{5}}$$

$$ \begin{aligned} & = \left({\left(0.5\right)}^{0.3}{\left(0.4\right)}^{0.2}{\left(0.9\right)}^{0.1}{\left(0.7\right)}^{0.2}{\left(0.6\right)}^{0.2}, {\left\{1-{\left(1-{\left(0.7\right)}^{3}\right)}^{0.3}{\left(1-{\left(0.8\right)}^{3}\right)}^{0.2}\right.}\right.\\ & \quad \left.{\left.{\left(1-{\left(0.2\right)}^{3}\right)}^{0.1}{\left(1-{\left(0.4\right)}^{3}\right)}^{0.2}{\left(1-{\left(0.5\right)}^{3}\right)}^{0.2}\right\}}^{1/3}\right)\end{aligned} $$

$$=(0.56257, 0.64371)$$

For $$\alpha =4$$

$$GIFWA{A}_{w}\left({m}_{1},{m}_{2}, {m}_{3}, {m}_{4}, {m}_{5}\right)={w}_{1}{m}_{1}\oplus {{w}_{2}m}_{2}\oplus {{w}_{3}m}_{3}\oplus {{w}_{4}m}_{4}\oplus {{w}_{5}m}_{5}$$

$$ \begin{aligned} & =\left({\left\{1-{\left(1-{\left(0.5\right)}^{4}\right)}^{0.3}{\left(1-{\left(0.4\right)}^{4}\right)}^{0.2}{\left(1-{\left(0.9\right)}^{4}\right)}^{0.1}\right.}\right.\\ & \quad \left.{\left.{\left(1-{\left(0.7\right)}^{4}\right)}^{0.2}{\left(1-{\left(0.6\right)}^{4}\right)}^{0.2}\right\}}^{1/4}, {\left(0.7\right)}^{0.3}{\left(0.8\right)}^{0.2}{\left(0.2\right)}^{0.1}{\left(0.4\right)}^{0.2}{\left(0.5\right)}^{0.1}\right)\end{aligned} $$

$$=\left(0.66249, 0.53022\right)$$

$$GIFWG{A}_{w}\left({m}_{1},{m}_{2}, {m}_{3}, {m}_{4}, {m}_{5}\right)={{m}_{1}}^{{w}_{1}}\otimes {{m}_{2}}^{{w}_{2}}\otimes {{m}_{3}}^{{w}_{3}}\otimes {{m}_{4}}^{{w}_{4}}\otimes {{m}_{5}}^{{w}_{5}}$$

$$ \begin{aligned} & =\left({\left(0.5\right)}^{0.3}{\left(0.4\right)}^{0.2}{\left(0.9\right)}^{0.1}{\left(0.7\right)}^{0.2}{\left(0.6\right)}^{0.2}, {\left\{1-{\left(1-{\left(0.7\right)}^{4}\right)}^{0.3}{\left(1-{\left(0.8\right)}^{4}\right)}^{0.2}\right. }\right.\\ & \quad \left.{\left. {\left(1-{\left(0.2\right)}^{4}\right)}^{0.1}{\left(1-{\left(0.4\right)}^{4}\right)}^{0.2}{\left(1-{\left(0.5\right)}^{4}\right)}^{0.2}\right\}}^{1/4}\right)\end{aligned} $$

$$=(0.56257, 0.65688)$$

**Table:** 

$\boldsymbol{\alpha }$	${\varvec{G}}{\varvec{I}}{\varvec{F}}{\varvec{W}}{\varvec{A}}{{\varvec{A}}}_{{\varvec{w}}}$	${\varvec{G}}{\varvec{I}}{\varvec{F}}{\varvec{W}}{\varvec{G}}{{\varvec{A}}}_{{\varvec{w}}}$
2	$\left(0.63262, 0.53022\right)$	$(0.56257, 0.62863)$
3	$\left(0.64739, 0.53022\right)$	$(0.56257, 0.64371)$
4	$\left(0.66249, 0.53022\right)$	$(0.56257, 0.65688)$

In the above table the aggregated values of GIFNs ${m}_{1},{m}_{2}, {m}_{3}, {m}_{4}$ and ${m}_{5}$, for different values of $\alpha$ by using GIFWAA and GIFWGA are presented.

### Theorem 4.6

(Idempotency) *Let all GIFNs* ${m}_{i}=\left({\mu }_{{m}_{i}},{\nu }_{{m}_{i}}\right), where i=\mathrm{1,2},\dots n$ *are such that* ${m}_{i}=m$, *then* $GIFWA{A}_{w}\left({m}_{1},{m}_{2},\dots {m}_{n}\right)$ *is* $m$.

### Proof

Let $$m=\left({\mu }_{m},{\nu }_{m}\right) and {m}_{i}=m,\mathrm{ where }i=\mathrm{1,2},\dots n$$, then

$$GIFWA{A}_{w}\left({m}_{1},{m}_{2},\dots {m}_{n}\right)=\left({\left\{1-{\prod }_{i=1}^{n}{\left(1-{\mu }_{{m}_{i}}^{\alpha }\right)}^{{w}_{i}}\right\}}^{1/\alpha}, {\prod }_{i=1}^{n}{\nu }_{{m}_{i}}^{{w}_{i}}\right)$$

$$=\left({\left\{1-{\prod }_{i=1}^{n}{\left(1-{\mu }_{m}^{\alpha }\right)}^{{w}_{i}}\right\}}^{1/\alpha}, {\prod }_{i=1}^{n}{\nu }_{m}^{{w}_{i}}\right)$$

$$=\left({\left\{1-{\left(1-{\mu }_{m}^{\alpha }\right)}^{\sum_{i=1}^{n}{w}_{i}}\right\}}^{1/\alpha}, {\nu }_{m}^{\sum_{i=1}^{n}{w}_{i}}\right)$$

$$=\left({\left\{1-\left(1-{\mu }_{m}^{\alpha }\right)\right\}}^{1/\alpha}, {\nu }_{m}\right)$$

$$=\left({\left\{1-1+{\mu }_{m}^{\alpha }\right\}}^{1/\alpha}, {\nu }_{m}\right)$$

$$=\left({\mu }_{m},{\nu }_{m}\right)=m\dots $$

### Theorem 4.7

(Boundedness) *Let*

$${m}^{-}=\left(\underset{i}{{\text{min}}}\left({\mu }_{{m}_{i}}\right), \underset{i}{{\text{max}}}\left({\nu }_{{m}_{i}}\right)\right)$$

*and*

$${m}^{+}=\left( \underset{i}{{\text{max}}}\left({\nu }_{{m}_{i}}\right), \underset{i}{{\text{min}}}\left({\mu }_{{m}_{i}}\right)\right).$$

*Then*,

$${m}^{-}\le GIFWA{A}_{w}\left({m}_{1},{m}_{2},\dots {m}_{n}\right)\le {m}^{+}.$$

### Proof

Since

$$GIFWA{A}_{w}\left({m}_{1},{m}_{2},\dots {m}_{n}\right)=\left({\left\{1-{\prod }_{i=1}^{n}{\left(1-{\mu }_{{m}_{i}}^{\alpha }\right)}^{{w}_{i}}\right\}}^{1/\alpha}, {\prod }_{i=1}^{n}{\nu }_{{m}_{i}}^{{w}_{i}}\right)$$

And for any $i$

$$\underset{i}{{\text{min}}}\left({\mu }_{{m}_{i}}\right)\le {\mu }_{{m}_{i}}\le \underset{i}{{\text{max}}}\left({\mu }_{{m}_{i}}\right)$$, $$\underset{i}{{\text{min}}}\left({\nu }_{{m}_{i}}\right)\le {\nu }_{{m}_{i}}\le \underset{i}{{\text{max}}}\left({\nu }_{{m}_{i}}\right)$$,

also,

$$\underset{i}{{\text{min}}}\left({\mu }_{{m}_{i}}^{\alpha }\right)\le {\mu }_{{m}_{i}}^{\alpha }\le \underset{i}{{\text{max}}}\left({\mu }_{{m}_{i}}^{\alpha }\right)$$ and $${\left(\underset{i}{{\text{min}}}\left({\nu }_{{m}_{i}}\right)\right)}^{{w}_{i}}\le {\nu }_{{m}_{i}}^{{w}_{i}}\le {\left(\underset{i}{{\text{max}}}\left({\nu }_{{m}_{i}}\right)\right)}^{{w}_{i}}$$.

Which implies,

$${\left\{1-{\prod }_{i=1}^{n}{\left(1-{\mu }_{{m}_{i}}^{\alpha }\right)}^{{w}_{i}}\right\}}^{1/\alpha}\ge {\left\{1-{\prod }_{i=1}^{n}{\left(1-\underset{i}{{\text{min}}}\left({\mu }_{{m}_{i}}^{\alpha }\right)\right)}^{{w}_{i}}\right\}}^{1/\alpha}$$

$$={\left\{1-{\left(1-\underset{i}{{\text{min}}}\left({\mu }_{{m}_{i}}^{\alpha }\right)\right)}^{\sum_{i=1}^{n}{w}_{i}}\right\}}^{1/\alpha}$$

$$=\underset{i}{{\text{min}}}\left({\mu }_{{m}_{i}}^{\alpha }\right)$$

$${\left\{1-{\prod }_{i=1}^{n}{\left(1-{\mu }_{{m}_{i}}^{\alpha }\right)}^{{w}_{i}}\right\}}^{1/\alpha}\le {\left\{1-{\prod }_{i=1}^{n}{\left(1-\underset{i}{{\text{max}}}\left({\mu }_{{m}_{i}}^{\alpha }\right)\right)}^{{w}_{i}}\right\}}^{1/\alpha}$$

$$={\left\{1-{\left(1-\underset{i}{{\text{max}}}\left({\mu }_{{m}_{i}}^{\alpha }\right)\right)}^{\sum_{i=1}^{n}{w}_{i}}\right\}}^{1/\alpha}$$

$$=\underset{i}{{\text{max}}}\left({\mu }_{{m}_{i}}^{\alpha }\right)$$

$${\prod }_{i=1}^{n}{\nu }_{{m}_{i}}^{{w}_{i}}\ge {\prod }_{i=1}^{n}{\left(\underset{i}{{\text{min}}}\left({\nu }_{{m}_{i}}\right)\right)}^{{w}_{i}}={\left(\underset{i}{{\text{min}}}\left({\nu }_{{m}_{i}}\right)\right)}^{\sum_{i=1}^{n}{w}_{i}}=\underset{i}{{\text{min}}}\left({\nu }_{{m}_{i}}\right)$$

and

$${\prod }_{i=1}^{n}{\nu }_{{m}_{i}}^{{w}_{i}}\le {\prod }_{i=1}^{n}{\left(\underset{i}{{\text{max}}}\left({\nu }_{{m}_{i}}\right)\right)}^{{w}_{i}}={\left(\underset{i}{{\text{max}}}\left({\nu }_{{m}_{i}}^{{w}_{i}}\right)\right)}^{\sum_{i=1}^{n}{w}_{i}}=\underset{i}{{\text{max}}}\left({\nu }_{{m}_{i}}\right)$$

Now, let

$$GIFWA{A}_{w}\left({m}_{1},{m}_{2},\dots {m}_{n}\right)=\left({\mu }_{m},{\nu }_{m}\right)=m$$, then

$$S\left(m\right)=\frac{{\left|{\Delta }_{m}\right|}^{1/\alpha}sgn\left({\Delta }_{m}\right)}{2}=\frac{{\left|{\mu }_{m}^{\alpha }-{\nu }_{m}^{\alpha }\right|}^{1/\alpha}sgn\left({\Delta }_{m}\right)}{2}\le \frac{{\left|\underset{i}{{\text{max}}}\left({\mu }_{{m}_{i}}^{\alpha }\right)-\underset{i}{{\text{min}}}\left({\nu }_{{m}_{i}}^{\alpha }\right)\right|}^{1/\alpha}sgn\left({\Delta }_{{m}^{+}}\right)}{2}=S\left({m}^{+}\right)$$

$$S\left(m\right)\le S\left({m}^{+}\right)$$

Also,

$$S\left(m\right)=\frac{{\left|{\Delta }_{m}\right|}^{1/\alpha}sgn\left({\Delta }_{m}\right)}{2}=\frac{{\left|{\mu }_{m}^{\alpha }-{\nu }_{m}^{\alpha }\right|}^{1/\alpha}sgn\left({\Delta }_{m}\right)}{2}\ge \frac{{\left|\underset{i}{{\text{max}}}\left({\mu }_{{m}_{i}}^{\alpha }\right)-\underset{i}{{\text{min}}}\left({\nu }_{{m}_{i}}^{\alpha }\right)\right|}^{1/\alpha}sgn\left({\Delta }_{{m}^{+}}\right)}{2}=S\left({m}^{-}\right)$$

$$S\left(m\right)\ge S\left({m}^{-}\right)$$

Now, we have three cases to discuss.

Case-1. if

$S\left(m\right)<S\left({m}^{+}\right)$ and $S\left(m\right)>S\left({m}^{-}\right)$, then by using Definition 3.2 Theorem 4.7 holds.

Case-2. if

$$S\left(m\right)=S\left({m}^{+}\right)$$

$$\frac{{\left|{\Delta }_{m}\right|}^{1/\alpha}sgn\left({\Delta }_{m}\right)}{2}=\frac{{\left|{\Delta }_{{m}^{+}}\right|}^{1/\alpha}sgn\left({\Delta }_{{m}^{+}}\right)}{2}$$

$$\frac{{\left|{\mu }_{m}^{\alpha }-{\nu }_{m}^{\alpha }\right|}^{1/\alpha}sgn\left({\Delta }_{m}\right)}{2}=\frac{{\left|\underset{i}{{\text{max}}}\left({\mu }_{{m}_{i}}^{\alpha }\right)-\underset{i}{{\text{min}}}\left({\nu }_{{m}_{i}}^{\alpha }\right)\right|}^{1/\alpha}sgn\left({\Delta }_{{m}^{+}}\right)}{2}$$

Which implies, that

$${\mu }_{m}^{\alpha }=\underset{i}{{\text{max}}}\left({\mu }_{{m}_{i}}^{\alpha }\right)$$

and

$${\nu }_{m}^{\alpha }=\underset{i}{{\text{min}}}\left({\nu }_{{m}_{i}}^{\alpha }\right)$$

Thus,

$$H\left(m\right)=\frac{{\left({\mu }_{m}^{\alpha }+{\nu }_{m}^{\alpha }\right)}^{1/\alpha}}{2}=H\left({m}^{+}\right)$$

Which implies, that

$$GIFWA{A}_{w}\left({m}_{1},{m}_{2},\dots {m}_{n}\right)={m}^{+}$$

Then, by using Definition 3.2, Theorem 4.7 holds

Case-3. Similarly,

$$ S\left( m \right) = S\left( {m^{ - } } \right),\;\;{\text{implies}}\;\;H\left( m \right) = H\left( {m^{ - } } \right) $$

Which implies, that

$$GIFWA{A}_{w}\left({m}_{1},{m}_{2},\dots {m}_{n}\right)={m}^{-}$$

Hence in all cases Theorem 4.7 must hold.

### Theorem 4.8

(Monotonicity) *Consider the sets of two collections of GIFNs denoted as* ${m}_{i}=\left({\mu }_{{m}_{i}},{\nu }_{{m}_{i}}\right)$, *and* ${m}^{*}=\left({\mu }_{{m}_{i}^{*}},{\nu }_{{m}_{i}^{*}}\right),$ *if* ${\mu }_{{m}_{i}}\le {\mu }_{{m}_{i}^{*}}$ and ${\nu }_{{m}_{i}}\ge {\nu }_{{m}_{i}^{*}}$ ,where $i=\mathrm{1,2},\dots n$, *then*

$$GIFWA{A}_{w}\left({m}_{1},{m}_{2},\dots {m}_{n}\right)\le GIFWA{A}_{w}\left({m}_{1}^{*},{m}_{2}^{*},\dots {m}_{n}^{*}\right).$$

### Proof

Since, ${\mu }_{{m}_{i}}\le {\mu }_{{m}_{i}^{*}}$ and ${\nu }_{{m}_{i}}\ge {\nu }_{{m}_{i}^{*}}$ for any $i$, we have

$${\left(1-{\mu }_{{m}_{i}}^{\alpha }\right)}^{{w}_{i}}\ge {\left(1-{\mu }_{{m}_{i}^{*}}^{\alpha }\right)}^{{w}_{i}}$$ and $${\nu }_{{m}_{i}}^{{w}_{i}}\le {\nu }_{{m}_{i}^{*}}^{{w}_{i}}$$

Implies,

$${\left\{1-{\prod }_{i=1}^{n}{\left(1-{\mu }_{{m}_{i}}^{\alpha }\right)}^{{w}_{i}}\right\}}^{1/\alpha}\le {\left\{1-{\prod }_{i=1}^{n}{\left(1-{\mu }_{{m}_{i}^{*}}^{\alpha }\right)}^{{w}_{i}}\right\}}^{1/\alpha}, {\prod }_{i=1}^{n}{\nu }_{{m}_{i}}^{{w}_{i}}\ge {\prod }_{i=1}^{n}{\nu }_{{m}_{i}^{*}}^{{w}_{i}}$$

Hence,

$${\left\{1-{\prod }_{i=1}^{n}{\left(1-{\mu }_{{m}_{i}}^{\alpha }\right)}^{{w}_{i}}\right\}}^{1/\alpha}-{\prod }_{i=1}^{n}{\nu }_{{m}_{i}}^{{w}_{i}}\le {\left\{1-{\prod }_{i=1}^{n}{\left(1-{\mu }_{{m}_{i}^{*}}^{\alpha }\right)}^{{w}_{i}}\right\}}^{1/\alpha}-{\prod }_{i=1}^{n}{\nu }_{{m}_{i}^{*}}^{{w}_{i}}$$

$$m=GIFWA{A}_{w}\left({m}_{1},{m}_{2},\dots {m}_{n}\right)=\left({\mu }_{m}, {\nu }_{m}\right)$$

$${m}^{*}=GIFWA{A}_{w}\left({m}_{1}^{*},{m}_{2}^{*},\dots {m}_{n}^{*}\right)=\left({\mu }_{{m}^{*}}, {\nu }_{{m}^{*}}\right)$$

Then from above, we have

$$\frac{{\left|{\mu }_{m}^{\alpha }-{\nu }_{m}^{\alpha }\right|}^{1/\alpha}sgn\left({\Delta }_{m}\right)}{2}\le \frac{{\left|\underset{i}{{\text{max}}}\left({\mu }_{{m}^{*}}^{\alpha }\right)-\underset{i}{{\text{min}}}\left({\nu }_{{m}^{*}}^{\alpha }\right)\right|}^{1/\alpha}sgn\left({\Delta }_{{m}^{*}}\right)}{2}$$

$$S\left(m\right)\le S\left({m}^{*}\right)$$

Now if $S\left(m\right)<S\left({m}^{*}\right)$, then by Definition 3.2$m<{m}^{*}$ i.e.

$$GIFWA{A}_{w}\left({m}_{1},{m}_{2},\dots {m}_{n}\right)<GIFWA{A}_{w}\left({m}_{1}^{*},{m}_{2}^{*},\dots {m}_{n}^{*}\right)$$

and if $S\left(m\right)=S\left({m}^{*}\right)$, then

$${\left\{1-{\prod }_{i=1}^{n}{\left(1-{\mu }_{{m}_{i}}^{\alpha }\right)}^{{w}_{i}}\right\}}^{1/\alpha}-{\prod }_{i=1}^{n}{\nu }_{{m}_{i}}^{{w}_{i}}={\left\{1-{\prod }_{i=1}^{n}{\left(1-{\mu }_{{m}_{i}^{*}}^{\alpha }\right)}^{{w}_{i}}\right\}}^{1/\alpha}-{\prod }_{i=1}^{n}{\nu }_{{m}_{i}^{*}}^{{w}_{i}}.$$

Then from the conditions ${\mu }_{{m}_{i}}\le {\mu }_{{m}_{i}^{*}}$ and ${\nu }_{{m}_{i}}\ge {\nu }_{{m}_{i}^{*}}$ for any $i$, we have

$${\left\{1-{\prod }_{i=1}^{n}{\left(1-{\mu }_{{m}_{i}}^{\alpha }\right)}^{{w}_{i}}\right\}}^{1/\alpha}={\left\{1-{\prod }_{i=1}^{n}{\left(1-{\mu }_{{m}_{i}^{*}}^{\alpha }\right)}^{{w}_{i}}\right\}}^{1/\alpha} and {\prod }_{i=1}^{n}{\nu }_{{m}_{i}}^{{w}_{i}}={\prod }_{i=1}^{n}{\nu }_{{m}_{i}^{*}}^{{w}_{i}}$$

therefore $H\left(m\right)=H\left({m}^{*}\right)$, implies $m={m}^{*}$, implies

$$GIFWA{A}_{w}\left({m}_{1},{m}_{2},\dots {m}_{n}\right)=GIFWA{A}_{w}\left({m}_{1}^{*},{m}_{2}^{*},\dots {m}_{n}^{*}\right)$$

Hence,

$$GIFWA{A}_{w}\left({m}_{1},{m}_{2},\dots {m}_{n}\right)\le GIFWA{A}_{w}\left({m}_{1}^{*},{m}_{2}^{*},\dots {m}_{n}^{*}\right).$$

Similarly, we can prove the following results for GIFWGA operator.

### Theorem 4.9

(Idempotency) *Let all GIFNs* ${m}_{i}=\left({\mu }_{{m}_{i}},{\nu }_{{m}_{i}}\right), where i=\mathrm{1,2},\dots n$ *are s.t* ${m}_{i}=m$, *then* $GIFWG{A}_{w}\left({m}_{1},{m}_{2},\dots {m}_{n}\right)$ *is* $m$.

### Theorem 4.10

(Boundedness) *Let all GIFNs* ${m}_{i}=\left({\mu }_{{m}_{i}},{\nu }_{{m}_{i}}\right), where i=\mathrm{1,2},\dots n$ *are s.t* ${m}_{i}=m$, *then*

$${m}^{-}\le GIFWG{A}_{w}\left({m}_{1},{m}_{2},\dots {m}_{n}\right)\le {m}^{+}$$

where

$${m}^{-}=\left(\underset{i}{{\text{min}}}\left({\mu }_{{m}_{i}}\right), \underset{i}{{\text{max}}}\left({\nu }_{{m}_{i}}\right)\right)$$

and

$${m}^{+}=\left( \underset{i}{{\text{max}}}\left({\nu }_{{m}_{i}}\right), \underset{i}{{\text{min}}}\left({\mu }_{{m}_{i}}\right)\right).$$

### Theorem 4.11

(Monotonicity) *Consider the sets of two collections of GIFNs denoted as* ${m}_{i}=\left({\mu }_{{m}_{i}},{\nu }_{{m}_{i}}\right)$ *and* ${m}^{*}=\left({\mu }_{{m}_{i}^{*}},{\nu }_{{m}_{i}^{*}}\right), where i=\mathrm{1,2},\dots n$, *if* ${\mu }_{{m}_{i}}\le {\mu }_{{m}_{i}^{*}}$ *and* ${\nu }_{{m}_{i}}\ge {\nu }_{{m}_{i}^{*}}$ *for any* $i$, *then*

$$GIFWG{A}_{w}\left({m}_{1},{m}_{2},\dots {m}_{n}\right)\le GIFWG{A}_{w}\left({m}_{1}^{*},{m}_{2}^{*},\dots {m}_{n}^{*}\right)$$

## Ordered weighted averaging aggregation operators with GIFNs

In this section , we present two aggregation operators, GIFOWAA operator and GIFOWGA operator. These operators are formulated in accordance with the operational principles governing GIFNs. We also delve into an exploration of key characteristics of these operators, providing a comprehensive understanding of their mathematical foundations within our research framework.

### Definition 5.1

Consider the set of GIFNs denoted as ${m}_{i}=\left({\mu }_{{m}_{i}},{\nu }_{{m}_{i}}\right)$, where $i=\mathrm{1,2},\dots n$, then the generalized intuitionistic fuzzy ordered weighted averaging aggregation (GIFOWAA) operator is a mapping $GIFOWAA: {m}^{n}\to m$ defined as

$$GIFOWA{A}_{\omega }\left({m}_{1},{m}_{2},\dots {m}_{n}\right)={\oplus }_{i=1}^{n}{\omega }_{i}{m}_{\delta (i)}$$

where $\omega ={({\omega }_{1},{\omega }_{2}\dots ,{\omega }_{n})}^{T}$ is the weight vector associated with GIFOWAA with ${\omega }_{i}\in \left[\mathrm{0,1}\right], such that \sum_{i=1}^{n}{\omega }_{i}=1$ and $\left(\delta \left(1\right), \delta \left(2\right),\dots ,\delta (n)\right)$ is a permutation of $\left(\mathrm{1,2},\dots ,n\right), s.t {m}_{\delta (i-1)}\ge {m}_{\delta (i)}$ for any i.

In particular case, when $\omega ={\left(\frac{1}{n}, \frac{1}{n},\dots , \frac{1}{n}\right)}^{T}$, then GIFOWAA operator reduces to generalized intuitionistic fuzzy averaging aggregation (GIFOAA) operator.

$$GIFAA\left({m}_{1},{m}_{2},\dots {m}_{n}\right)=\frac{1}{n}{\oplus }_{i=1}^{n}{m}_{i}$$

Similar to Theorem 4.2, we have the following result.

### Theorem 5.2

*Consider the set of GIFNs denoted as* ${m}_{i}=\left({\mu }_{{m}_{i}},{\nu }_{{m}_{i}}\right)$, *where* $i=\mathrm{1,2},\dots n$, *then the aggregated value of *${m}_{i}$* by using GIFOWAA operator is also a GIFN, where*

$$GIFOWA{A}_{\omega }\left({m}_{1},{m}_{2},\dots {m}_{n}\right)=\left({\left\{1-{\prod }_{i=1}^{n}{\left(1-{\mu }_{{m}_{\delta (i)}}^{\alpha }\right)}^{{\omega }_{i}}\right\}}^{1/\alpha}, {\prod }_{i=1}^{n}{\nu }_{{m}_{\delta (i)}}^{{\omega }_{i}}\right).$$

Where $\omega ={({\omega }_{1},{\omega }_{2}\dots ,{\omega }_{n})}^{T}$ is the weight vector associated with GIFOWAA with ${\omega }_{i}\in \left[\mathrm{0,1}\right],\mathrm{ such that} \sum_{i=1}^{n}{\omega }_{i}=1$ and $\left(\delta \left(1\right), \delta \left(2\right),\dots ,\delta (n)\right)$ is a permutation of $\left(\mathrm{1,2},\dots ,n\right), s.t {m}_{\delta (i-1)}\ge {m}_{\delta (i)}$ for any $i$ and $\alpha =n or \frac{1}{n} and n\in {\mathbb{N}},n>0$

In particular if ${\mu }_{{m}_{i}}^{\alpha }=1-{\nu }_{{m}_{i}}^{\alpha }$, then

$$GIFOWA{A}_{\omega }\left({m}_{1},{m}_{2},\dots {m}_{n}\right)=\left({\left\{1-{\prod }_{i=1}^{n}{\left(1-{\mu }_{{m}_{\delta (i)}}^{\alpha }\right)}^{{\omega }_{i}}\right\}}^{1/\alpha}, {\left\{{\prod }_{i=1}^{n}{\left(1-{\mu }_{{m}_{\delta (i)}}^{\alpha }\right)}^{{\omega }_{i}}\right\}}^{1/\alpha}\right).$$

### Definition 5.3

Consider the set of GIFNs denoted as ${m}_{i}=\left({\mu }_{{m}_{i}},{\nu }_{{m}_{i}}\right)$, where $i=\mathrm{1,2},\dots n$, then the generalized intuitionistic fuzzy ordered weighted geometric aggregation (GIFOWGA) operator is a mapping $GIFOWGA: {m}^{n}\to m$ defined as

$$GIFOWG{A}_{\omega }\left({m}_{1},{m}_{2},\dots {m}_{n}\right)={\otimes}_{i=1}^{n}{\left({m}_{\delta (i)}\right)}^{{\omega }_{i}}$$

where $\omega ={({\omega }_{1},{\omega }_{2}\dots ,{\omega }_{n})}^{T}$ is the weight vector associated with GIFOWGA with ${\omega }_{i}\in \left[\mathrm{0,1}\right],\mathrm{ such that} \sum_{i=1}^{n}{\omega }_{i}=1$ and $\left(\delta \left(1\right), \delta \left(2\right),\dots ,\delta (n)\right)$ is a permutation of $\left(\mathrm{1,2},\dots ,n\right), s.t {m}_{\delta (i-1)}\ge {m}_{\delta (i)}$ for any $i$.

In particular case, when $\omega ={\left(\frac{1}{n}, \frac{1}{n},\dots , \frac{1}{n}\right)}^{T}$, then GIFOWGA operator reduces to generalized intuitionistic fuzzy averaging aggregation (GIFGA) operator.

$$GIFGA\left({m}_{1},{m}_{2},\dots {m}_{n}\right)={\left({\otimes}_{i=1}^{n}{m}_{\delta (i)}\right)}^\frac{1}{n}$$

Similar to Theorem 4.4, we have the following result.

### Theorem 5.4

*Consider the set of GIFNs denoted as* ${m}_{i}=\left({\mu }_{{m}_{i}},{\nu }_{{m}_{i}}\right)$, *where* $i=\mathrm{1,2},\dots n$, *then the aggregated value of *${m}_{i}$* by using GIFOWGA operator is also a GIFN, where*

$$GIFOWG{A}_{\omega }\left({m}_{1},{m}_{2},\dots {m}_{n}\right)=\left({\prod }_{i=1}^{n}{\mu }_{{m}_{\delta (i)}}^{{\omega }_{i}}, {\left\{1-{\prod }_{i=1}^{n}{\left(1-{\nu }_{{m}_{\delta (i)}}^{\alpha }\right)}^{{\omega }_{i}}\right\}}^{1/\alpha}\right)$$

where $\omega ={({\omega }_{1},{\omega }_{2}\dots ,{\omega }_{n})}^{T}$ is the weight vector associated with GIFOWGA with ${\omega }_{i}\in \left[\mathrm{0,1}\right],\mathrm{ such that }\sum_{i=1}^{1}{\omega }_{i}=1$ and $\left(\delta \left(1\right), \delta \left(2\right),\dots ,\delta (n)\right)$ is a permutation of $\left(\mathrm{1,2},\dots ,n\right), s.t {m}_{\delta (i-1)}\ge {m}_{\delta (i)}$ for any $i$ and $\alpha =n or \frac{1}{n} and n\in {\mathbb{N}},n>0$

In particular if ${\mu }_{{m}_{i}}^{\alpha }=1-{\nu }_{{m}_{i}}^{\alpha }$ , then

$$GIFWG{A}_{\omega }\left({m}_{1},{m}_{2},\dots {m}_{n}\right)=\left({\prod }_{i=1}^{n}{\mu }_{{m}_{\delta (i)}}^{{\omega }_{i}}, {\left\{1-{\prod }_{i=1}^{n}{\mu }_{{m}_{\delta (i)}}^{\alpha {\omega }_{i}}\right\}}^{1/\alpha}\right)$$

Similar to GIFWAA and GIFWGA operators, we have the following properties for GIFOWAA and GIFOWGA operators.

### Theorem 5.5

(Idempotency) *Let all GIFNs* ${m}_{i}=\left({\mu }_{{m}_{i}},{\nu }_{{m}_{i}}\right) ,\mathrm{ where }i=\mathrm{1,2},\dots n$ *are s.t* ${m}_{i}=m$, *then*

$$GIFOWA{A}_{w}\left({m}_{1},{m}_{2},\dots {m}_{n}\right)=m\dots ,$$

$$GIFOWG{A}_{w}\left({m}_{1},{m}_{2},\dots {m}_{n}\right)=m\dots $$

### Theorem 5.6

(Boundedness) Let all GIFNs ${m}_{i}=\left({\mu }_{{m}_{i}},{\nu }_{{m}_{i}}\right),\mathrm{ where }i=\mathrm{1,2},\dots n$ are s.t ${m}_{i}=m$, *then*

$${m}^{-}\le GIFOWA{A}_{w}\left({m}_{1},{m}_{2},\dots {m}_{n}\right)\le {m}^{+}$$

$${m}^{-}\le GIFOWG{A}_{w}\left({m}_{1},{m}_{2},\dots {m}_{n}\right)\le {m}^{+}$$

*where*,

$${m}^{-}=\left(\underset{i}{{\text{min}}}\left({\mu }_{{m}_{i}}\right), \underset{i}{{\text{max}}}\left({\nu }_{{m}_{i}}\right)\right)$$

*and*

$${m}^{+}=\left( \underset{i}{{\text{max}}}\left({\nu }_{{m}_{i}}\right), \underset{i}{{\text{min}}}\left({\mu }_{{m}_{i}}\right)\right).$$

### Theorem 5.7

(Monotonicity) *Consider the sets of two collections of GIFNs denoted as* ${m}_{i}=\left({\mu }_{{m}_{i}},{\nu }_{{m}_{i}}\right)$ *and* ${m}^{*}=\left({\mu }_{{m}_{i}^{*}},{\nu }_{{m}_{i}^{*}}\right), where i=\mathrm{1,2},\dots n$, *if* ${\mu }_{{m}_{i}}\le {\mu }_{{m}_{i}^{*}}$ *and* ${\nu }_{{m}_{i}}\ge {\nu }_{{m}_{i}^{*}}$ *for any* $i$, *then*

$$GIFOWA{A}_{w}\left({m}_{1},{m}_{2},\dots {m}_{n}\right)\le GIFOWA{A}_{w}\left({m}_{1}^{*},{m}_{2}^{*},\dots {m}_{n}^{*}\right),$$

$$GIFOWG{A}_{w}\left({m}_{1},{m}_{2},\dots {m}_{n}\right)\le GIFOWG{A}_{w}\left({m}_{1}^{*},{m}_{2}^{*},\dots {m}_{n}^{*}\right).$$

Additionally the GIFOWAA and GIFOWGA operators also possess the following desirable properties.

### Theorem 5.8

(Commutativity) *Let* ${m}_{i}=\left({\mu }_{{m}_{i}},{\nu }_{{m}_{i}}\right)$ *and* ${m}_{i}^{*}=\left({\mu }_{{m}_{i}^{*}},{\nu }_{{m}_{i}^{*}}\right),\mathrm{ where }i=\mathrm{1,2},\dots n$ *be two collections of GIFNs. Then for any permutation* $\left({m}_{1}^{*},{m}_{2}^{*},\dots {m}_{n}^{*}\right)$ *of* $\left({m}_{1},{m}_{2},\dots {m}_{n}\right)$

(i) $$GIFOWA{A}_{w}\left({m}_{1},{m}_{2},\dots {m}_{n}\right)=GIFOWA{A}_{w}\left({m}_{1}^{*},{m}_{2}^{*},\dots {m}_{n}^{*}\right)$$,
(ii) $$GIFOWG{A}_{w}\left({m}_{1},{m}_{2},\dots {m}_{n}\right)=GIFOWG{A}_{w}\left({m}_{1}^{*},{m}_{2}^{*},\dots {m}_{n}^{*}\right)$$.

### Proof

Let $GIFOWA{A}_{\omega }\left({m}_{1},{m}_{2},\dots {m}_{n}\right)={\oplus }_{i=1}^{n}{\omega }_{i}{m}_{\delta (i)}$ and $GIFOWA{A}_{\omega }\left({m}_{1}^{*},{m}_{2}^{*},\dots {m}_{n}^{*}\right)={\oplus }_{i=1}^{n}{\omega }_{i}{m}_{\delta (i)}^{*}$, since $\left({m}_{1}^{*},{m}_{2}^{*},\dots {m}_{n}^{*}\right)$ is any permutation of $\left({m}_{1},{m}_{2},\dots {m}_{n}\right)$, so ${m}_{\delta \left(i\right)}={m}_{\delta \left(i\right)}^{*},\mathrm{where }(i=\mathrm{1,2},\dots n)$.

Thus Theorem 5.8(i) holds. Similarly it can be prove that Theorem 5.8(ii) also holds.

From Theorem 5.8 it can be seen that for both GIFOWAA and GIFOWGA operators possess the Commutativity property but GIFWAA and GIFWGA operators do not possess it.

## MCDM technique by utilizing GIFWAA and GIFWGA

This section introduces a MCDM that leverages the GIFWAA and GIFWGA operators. This technique is developed to address MCDM challenges where criteria values and weights are represented as GIFNs. Initially, the mathematical framework of the MCDM problem incorporating Generalized Intuitionistic Fuzzy information is delineated. Subsequently, the proposed technique is elucidated, demonstrating its enhanced efficacy in addressing such complex MCDM scenarios. To illustrate the practical application and effectiveness of the proposed technique, a numerical example centered around the selection of a prominent tech company is presented. This example serves to underscore the pragmatic utility and relevance of our devised approach within real-world decision-making contexts.

### Analytical representation of the MCDM problem

In a given multi-criteria decision making (MCDM) scenario within the context of Generalized Intuitionistic Fuzzy framework, let us consider a collection of feasible alternatives $A=\left\{{A}_{1},{A}_{2},\dots ,{A}_{m}\right\}$, and a finite set of criteria $C=\left\{{C}_{1}, {C}_{2}, \dots , {C}_{n}\right\}$ that are established by the expert $\xi$. Let $w={({w}_{1},{w}_{2}\dots ,{w}_{n})}^{T}$ represent the associated weight vector for the criteria, where ${w}_{j}> 0$ and the sum of all weights $\sum_{j=1}^{n}{w}_{j}=1$. The expert $\xi$ evaluates the available alternatives ${A}_{i}(i = 1, 2, 3, \dots , m)$ in relation to the criterion ${C}_{j}(j = 1, 2, 3, \dots , n)$ under consideration. The evaluation is expressed through a Generalized Intuitionistic Fuzzy Decision Matrix (GIFDM) denoted as $Q={\left({m}_{ij}\right)}_{m\times n}$, where each element ${m}_{ij} = ({\mu }_{ij}, {\nu }_{ij})$ corresponds to a GIFN provided by the expert $\xi$. Here, ${\mu }_{ij}$ represents the degree to which alternative ${A}_{i}$ satisfies criterion ${C}_{j}$, and ${\nu }_{ij}$ represents the degree to which alternative ${A}_{i}$ dissatisfies criterion ${C}_{j}$. Consequently, the MCDM problem is formalized in the following matrix representation:

$$Q = \left( {\begin{array}{*{20}c} {{(}\mu_{11} {,}\nu_{{{11}}} {)}} & {{(}\mu_{12} {,}\nu_{{{12}}} {)}} & \cdots & {{(}\mu_{1n} {,}\nu_{{{\text{1n}}}} {)}} \\ {{(}\mu_{21} {,}\nu_{{{21}}} {)}} & {{(}\mu_{21} {,}\nu_{{{21}}} {)}} & \cdots & {{(}\mu_{2n} {,}\nu_{{{\text{2n}}}} {)}} \\ \vdots & \vdots & \ddots & \vdots \\ {{(}\mu_{m1} {,}\nu_{{{\text{m1}}}} {)}} & {{(}\mu_{m2} {,}\nu_{{{\text{m2}}}} {)}} & \cdots & {{(}\mu_{mn} {,}\nu_{{{\text{mn}}}} {)}} \\ \end{array} } \right).$$

### Decision-making technique based on GIFWAA and GIFWGA operators

The subsequent delineated steps for decision-making offer a systematic approach to effectively address the aforementioned MCDM problem with Generalized Intuitionistic Fuzzy information.

Step 1. Construction of Generalized Intuitionistic Fuzzy Decision Matrix $Q={\left({m}_{ij}\right)}_{m\times n}$

Step 2. Derive the collective comprehensive preference value, denoted as ${m}_{i} (i = 1, 2, \dots ,m)$, for each alternative ${A}_{i}(i = 1, 2, 3, \dots , m)$ with respect to the criteria ${C}_{j}(j = 1, 2, 3, \dots , n)$ by using the GIFWAA operator

$${m}_{i}=GIFWA{A}_{w}\left({m}_{i1},{m}_{i2},\dots {m}_{in}\right)={\oplus }_{j=1}^{n}{w}_{j}{m}_{ij}$$

$$=\left({\left\{1-{\prod }_{j=1}^{n}{\left(1-{\mu }_{{m}_{ij}}^{\alpha }\right)}^{{w}_{j}}\right\}}^{1/\alpha}, {\prod }_{j=1}^{n}{\nu }_{{m}_{ij}}^{{w}_{j}}\right)$$

or by using the GIFWGA operator

$${m}_{i}=GIFWG{A}_{w}\left({m}_{i1},{m}_{i2},\dots {m}_{in}\right)={\otimes}_{j=1}^{n}{\left({m}_{ij}\right)}^{{w}_{j}}$$

$$=\left({\prod }_{j=1}^{n}{\mu }_{{m}_{ij}}^{{w}_{j}}, {\left\{1-{\prod }_{j=1}^{n}{\left(1-{\nu }_{{m}_{ij}}^{\alpha }\right)}^{{w}_{j}}\right\}}^{1/\alpha}\right).$$

Where $w={\left({w}_{1},{w}_{2}\dots ,{w}_{n}\right)}^{T}$ is the weight vector s.t ${w}_{j}\in \left[\mathrm{0,1}\right], \left(i=\mathrm{1,2},\dots n\right)$ and

$$\sum_{j=1}^{1}{w}_{j}=1$$ and $$\alpha =n or \frac{1}{n} and n\in {\mathbb{N}},n>0.$$

Step 3. Calculate the score functions $S\left({m}_{i}\right),$ from Definition 3.2 of the collective comprehensive preference values ${m}_{i}$ to rank the alternatives.

Step 4. Using the score functions, prioritize the alternatives to select the optimal choice from among them.

**Figure 2:**
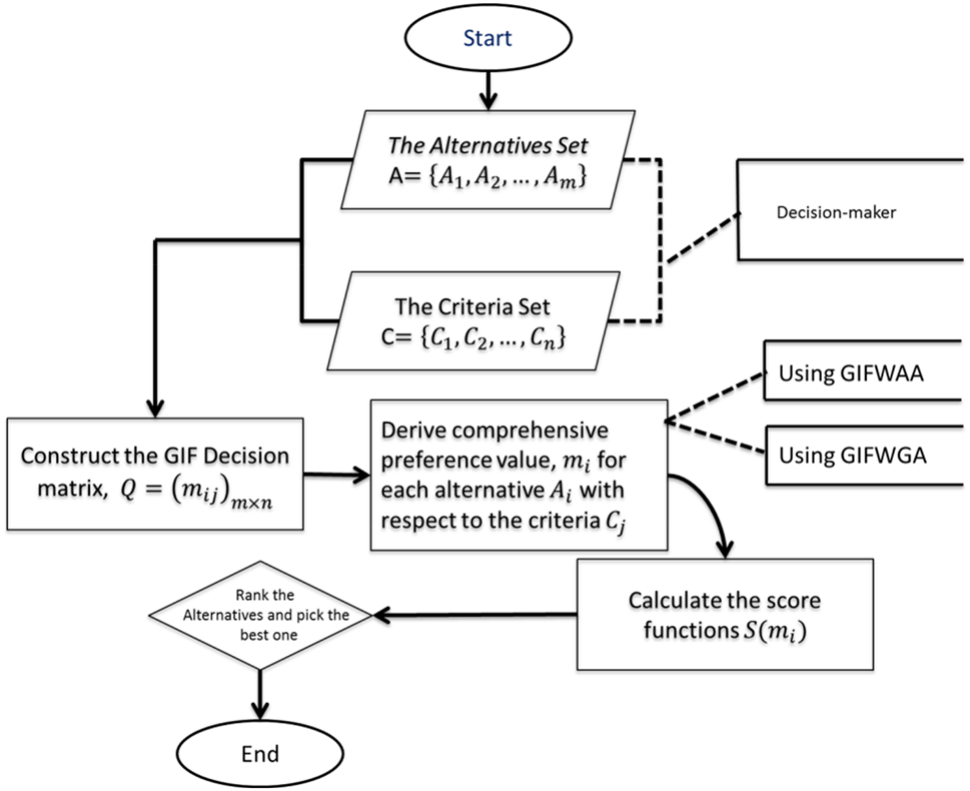
The flowchart of proposed strategy.

This procedural framework offers a comprehensive guide for efficiently addressing the MCDM problem while incorporating Generalized Intuitionistic Fuzzy information. Figure 2 depicts the flowchart of proposed multiple criteria decision-making strategy.

Here is Algorithm 1 that depicts the pseudo code representation of proposed MCDM strategy

**Algorithm 1:**
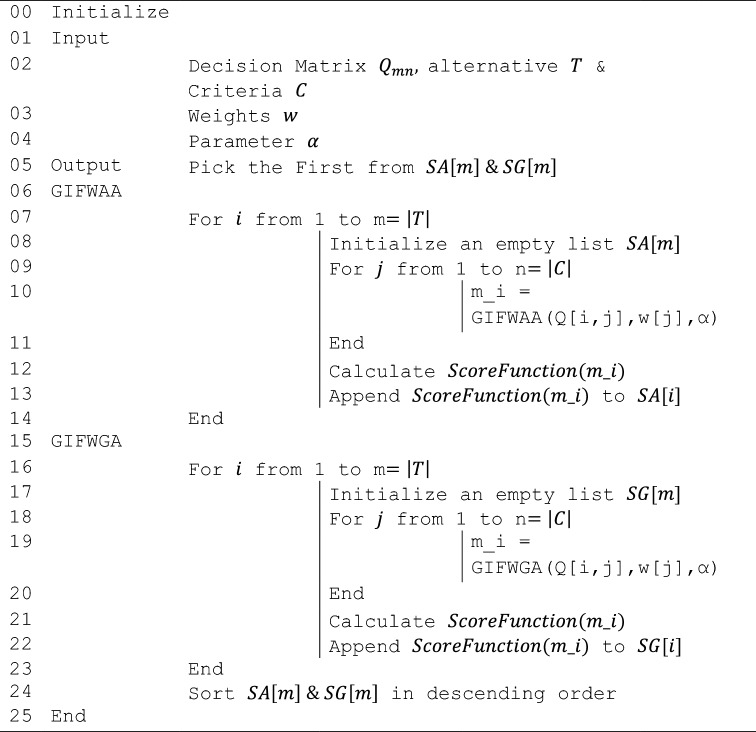
.

.

Next, we provide an Application of Proposed MCDM in Tech Industry to demonstrate the proposed approach:

### Application of proposed MCDM in tech industry evaluation for startup success

In this section, we adapted the data from an example from Akram^41^ to illustrate the multiple criteria decision-making with a generalized intuitionistic fuzzy environment.

The technology and electronics industry holds significant importance in driving economic and societal progress across diverse nations. The art of designing innovative gadgets, software, and digital solutions in alignment with current technological trends stands as a pivotal driver for leading sectors. The potential for technology industries to catalyze long-term growth is influenced not only by investors' metrics but also by the excellence of their products. Technological trends evolve over time and vary across regions, reflecting the unique characteristics of each locale. A recent graduate in computer science is preparing to launch her tech startup in the city. Given the paramount significance of cutting-edge technology, she evaluates five tech companies denoted as ${{\varvec{T}}}_{{\varvec{i}}}({\varvec{i}}=1,2,3,\dots ,5)$ that has a strong presence in the market. Seeking strategic guidance, she seeks the expertise of an analytical technology consultant, denoted as ${\varvec{\xi}}$, to swiftly and effectively determine the optimal choice among the available industries. The consultant assesses five tech companies across four criteria, identified as ${{\varvec{C}}}_{{\varvec{j}}}({\varvec{j}}\boldsymbol{ }=\boldsymbol{ }1,\boldsymbol{ }2,\boldsymbol{ }\dots ,4)$, which include: C1: Product Durability, C2: Product Price, C4: Functionality and User Experience, and C5: Technological Innovation. These criteria are assigned corresponding weight vectors,${{\varvec{w}}}_{{\varvec{j}}}={\left(0.4,\boldsymbol{ }0.3,\boldsymbol{ }0.2,\boldsymbol{ }0.1\right)}^{{\varvec{T}}}$, as specified by the decision maker. The decision matrix ${\varvec{Q}}={({{\varvec{m}}}_{{\varvec{i}}{\varvec{j}}})}_{5\times 4}$ is presented in Table 3, encapsulating the generalized intuitionistic fuzzy information.

**Table 3:** Generalized intuitionistic fuzzy decision matrix (GIFDM) of the analytical technology consultant.

	**C**_**1**_	**C**_**2**_	**C**_**3**_	**C**_**4**_
${\varvec{T}}$_1_	$\left(\mathrm{0.5,0.6}\right)$	$\left(\mathrm{0.9,0.2}\right)$	$\left(\mathrm{0.3,0.6}\right)$	$\left(\mathrm{0.7,0.6}\right)$
${\varvec{T}}$_2_	$\left(\mathrm{0.7,0.4}\right)$	$\left(\mathrm{0.2,0.6}\right)$	$\left(\mathrm{0.1,0.9}\right)$	$\left(\mathrm{0.5,0.8}\right)$
${\varvec{T}}$_3_	$\left(\mathrm{0.3,0.9}\right)$	$\left(\mathrm{0.3,0.4}\right)$	$\left(\mathrm{0.8,0.5}\right)$	$\left(\mathrm{0.6,0.8}\right)$
${\varvec{T}}$_4_	$\left(\mathrm{0.4,0.6}\right)$	$\left(\mathrm{0.7,0.3}\right)$	$\left(\mathrm{0.4,0.7}\right)$	$\left(\mathrm{0.5,0.5}\right)$
${\varvec{T}}$_5_	$\left(\mathrm{0.3,0.2}\right)$	$\left(\mathrm{0.8,0.4}\right)$	$\left(\mathrm{0.5,0.6}\right)$	$\left(\mathrm{0.3,0.8}\right)$

The data in the Table 1 shows the fuzzy information by the consultant ${\varvec{\xi}}$ in the form of GIFN_B_s. From the data in the above table it can be clearly seen that these are not IFNs, but are GIFN_B_s.

To choose the most profitable and suitable tech companies among all the companies ${T}_{i}(i = 1, 2, 3, \dots , 5)$, we apply the GIFWAA and GIFWGA operator. The MCDM steps for $\alpha = 2$ can be described as below:

Step 1. Construction of Generalized Intuitionistic Fuzzy Decision Matrix $Q={\left({m}_{ij}\right)}_{m\times n}$

Step 2. Derive the collective comprehensive preference value ${m}_{i} (i = 1, 2, \dots ,5)$ of the companies ${T}_{i}(i = 1, 2, 3, \dots , 5)$ with respect to the criteria ${C}_{j}(j = 1, 2, \dots , 4)$ by using the GIFWAA operator which is as follows:

$${m}_{1}=\left(\mathrm{0.7093,0.4315}\right), {m}_{2}=\left(0.5180, 0.5694\right), {m}_{3}=\left(0.5198, 0.6200\right), {m}_{4}=\left(0.5338, 0.4935\right) and {m}_{5}=\left(0.5806, 0.3523\right)$$

Step 3. Calculate the score functions $S({m}_{i})$, from Definition 3.2 of the collective comprehensive preference values ${m}_{i}(i = 1, 2, \dots ,5)$ as follows.

$$S\left({m}_{1}\right)=0.2815S\left({m}_{2}\right)=-0.1182S\left({m}_{3}\right)=-0.1690S\left({m}_{4}\right)=0.1017S\left({m}_{5}\right)=0.2307.$$

Step 4. Using the score functions, prioritize the companies as

$${T}_{1}\succ {T}_{5}\succ {T}_{4}\succ {T}_{2}\succ {T}_{3}$$

According to the ranking, it is concluded that ${T}_{1}$ is the most profitable and

suitable tech company among all.

By using GIFWGA operator. The MCDM steps, for $\alpha = 2$, can be described as below:

Step 1. Construction of Generalized Intuitionistic Fuzzy Decision Matrix $Q={\left({m}_{ij}\right)}_{m\times n}$

Step 2. Derive the collective comprehensive preference value ${m}_{i} (i = 1, 2, \dots ,5)$ of the companies ${T}_{i}(i = 1, 2, 3, \dots , 5)$ with respect to the criteria ${C}_{j}(j = 1, 2, \dots , 4)$ by using the GIFWGA operator which is as follows:

$${m}_{1}=\left(\mathrm{0.5569,0.5265}\right), {m}_{2}=\left(0.3150, 0.6867\right), {m}_{3}=\left(0.3912, 0.7640\right), {m}_{4}=\left(0.4838, 0.5563\right) and {m}_{5}=\left(0.4459, 0.4785\right)$$

Step 3. Calculate the score functions $S({m}_{i})$, from Definition 3.2 of the collective comprehensive preference values ${m}_{i}(i = 1, 2, \dots ,5)$ as follows.

$$S\left({m}_{1}\right)=0.0908$$ , $$S\left({m}_{2}\right)=-0.3051$$ , $$S\left({m}_{3}\right)=-0.3281$$ , $$S\left({m}_{4}\right)=-0.1373$$ , $$S\left({m}_{5}\right)=-0.0868$$

Step 4. Using the score functions, prioritize the companies as

$${T}_{1}\succ {T}_{5}\succ {T}_{4}\succ {T}_{2}\succ {T}_{3}$$

According to the ranking, it is concluded that ${T}_{1}$ is the most profitable and suitable tech company among all.

#### Analyzing the impact of parameter $\boldsymbol{\alpha }$ on decision-making outcomes

To comprehend the influence of parameter $\alpha$ on decision-making results, we explore different values of α to rank the alternatives. The collective comprehensive preference value ${m}_{i} (i = 1, 2, \dots ,5)$ of the companies ${T}_{i}(i = 1, 2, 3, \dots , 5)$ with respect to the criteria ${C}_{j}(j = 1, 2, \dots , 4)$ , score function outcomes for the alternatives ${T}_{i}(i = 1, 2, 3, \dots , 5)$ and the ranking order have been evaluated.

Preference value ${m}_{i} (i = 1, 2, \dots ,5)$ of the companies ${T}_{i}(i = 1, 2, 3, \dots , 5)$ with respect to the criteria ${C}_{j}(j = 1, 2, \dots , 4)$, by using the GIFWAA are presented in the above Table 4 for different values of α.

**Table 4:** Preference value ${m}_{i} (i = 1, 2, \dots ,5)$ by using GIFWAA.

Α	Preference value ${{\varvec{m}}}_{{\varvec{i}}}({\varvec{i}}=1,2,\dots ,5)$ by using the GIFWAA
2	${m}_{1}=\left(\mathrm{0.7093,0.4315}\right), {m}_{2}=\left(0.5180, 0.5694\right), {m}_{3}=\left(0.5198, 0.6200\right),$ ${m}_{4}=\left(0.5338, 0.4935\right) and {m}_{5}=\left(0.5806, 0.3523\right)$
3	${m}_{1}=\left(\mathrm{0.7300,0.4315}\right), {m}_{2}=\left(0.5518, 0.5694\right), {m}_{3}=\left(0.5546, 0.6200\right),$ ${m}_{4}=\left(0.5473, 0.4935\right) and {m}_{5}=\left(0.6087, 0.3523\right)$
4	${m}_{1}=\left(\mathrm{0.7478,0.4315}\right), {m}_{2}=\left(0.5762, 0.5694\right), {m}_{3}=\left(0.5854, 0.6200\right),$ ${m}_{4}=\left(0.5611, 0.4935\right) and {m}_{5}=\left(0.6330, 0.3523\right)$
5	${m}_{1}=\left(\mathrm{0.7631,0.4315}\right), {m}_{2}=\left(0.5941, 0.5694\right), {m}_{3}=\left(0.6113, 0.6200\right),$ ${m}_{4}=\left(0.5744, 0.4935\right) and {m}_{5}=\left(0.6533, 0.3523\right)$
6	${m}_{1}=\left(\mathrm{0.7760,0.4315}\right), {m}_{2}=\left(0.6077, 0.5694\right), {m}_{3}=\left(0.6324, 0.6200\right),$ ${m}_{4}=\left(\mathrm{0.5866,0.4935}\right) and {m}_{5}=\left(0.6699, 0.3523\right)$
7	${m}_{1}=\left(\mathrm{0.7870,0.4315}\right), {m}_{2}=\left(0.6183, 0.5694\right), {m}_{3}=\left(0.6496, 0.6200\right),$ ${m}_{4}=\left(\mathrm{0.5975,0.4935}\right) and {m}_{5}=\left(0.6836, 0.3523\right)$
8	${m}_{1}=\left(\mathrm{0.7963,0.4315}\right), {m}_{2}=\left(0.6269, 0.5694\right), {m}_{3}=\left(0.6639, 0.6200\right),$ ${m}_{4}=\left(\mathrm{0.6070,0.4935}\right) and {m}_{5}=\left(0.6949, 0.3523\right)$
9	${m}_{1}=\left(\mathrm{0.8044,0.4315}\right), {m}_{2}=\left(0.6339, 0.5694\right), {m}_{3}=\left(0.6758, 0.6200\right),$ ${m}_{4}=\left(\mathrm{0.6152,0.4935}\right) and {m}_{5}=\left(0.7044, 0.3523\right)$
10	${m}_{1}=\left(\mathrm{0.8113,0.4315}\right), {m}_{2}=\left(0.6398, 0.5694\right), {m}_{3}=\left(0.6859, 0.6200\right),$ ${m}_{4}=\left(\mathrm{0.6224,0.4935}\right) and {m}_{5}=\left(0.7125, 0.3523\right)$

The score function outcomes for the alternatives ${T}_{i}(i = 1, 2, 3, \dots , 5)$ and the ranking order have been evaluated for the corresponding values ${m}_{i} (i = 1, 2, \dots ,5)$ by using the GIFWAA are presented in the above Table 5 for different values of α.

**Table 5:** Ranking orders by using GIFWAA.

Α	score functions $\mathbf{S}({\mathbf{m}}_{\mathbf{i}})$	Ranking orders by using GIFWAA
2	$S\left({m}_{1}\right)=0.2815$, $S\left({m}_{2}\right)=-0.1182$, $S\left({m}_{3}\right)=-0.1690$ , $S\left({m}_{4}\right)=0.1017$,$S\left({m}_{5}\right)=0.2308$	${T}_{1}\succ {T}_{5}\succ {T}_{4}\succ {T}_{2}\succ {T}_{3}$
3	$S\left({m}_{1}\right)=0.3379$, $S\left({m}_{2}\right)=-0.1275$, $S\left({m}_{3}\right)=-0.2038$ , $S\left({m}_{4}\right)=0.1762$,$S\left({m}_{5}\right)=0.2833$	${T}_{1}\succ {T}_{5}\succ {T}_{4}\succ {T}_{2}\succ {T}_{3}$
4	$S\left({m}_{1}\right)=0.3631$, $S\left({m}_{2}\right)=0.1336$ , $S\left({m}_{3}\right)=-0.2087$ , $S\left({m}_{4}\right)=0.2233$,$S\left({m}_{5}\right)=0.3086$	${T}_{1}\succ {T}_{5}\succ {T}_{4}\succ {T}_{2}\succ {T}_{3}$
5	$S\left({m}_{1}\right)=0.3770$, $S\left({m}_{2}\right)=0.2133$ , $S\left({m}_{3}\right)=-0.1812$ , $S\left({m}_{4}\right)=0.2531$,$S\left({m}_{5}\right)=0.3236$	${T}_{1}\succ {T}_{5}\succ {T}_{4}\succ {T}_{2}\succ {T}_{3}$
6	$S\left({m}_{1}\right)=0.3861$, $S\left({m}_{2}\right)=0.2517$, $S\left({m}_{3}\right)=0.2194$, $S\left({m}_{4}\right)=0.2726$,$S\left({m}_{5}\right)=0.3338$	${T}_{1}\succ {T}_{5}\succ {T}_{4}\succ {T}_{2}\succ {T}_{3}$
7	$S\left({m}_{1}\right)=0.3926$, $S\left({m}_{2}\right)=0.2748$, $S\left({m}_{3}\right)=0.2706$, $S\left({m}_{4}\right)=0.2860$,$S\left({m}_{5}\right)=0.3413$	${T}_{1}\succ {T}_{5}\succ {T}_{4}\succ {T}_{2}\succ {T}_{3}$
8	$S\left({m}_{1}\right)=0.3978$, $S\left({m}_{2}\right)=0.2900$, $S\left({m}_{3}\right)=0.2979$, $S\left({m}_{4}\right)=0.2956$,$S\left({m}_{5}\right)=0.3473$	${T}_{1}\succ {T}_{5}\succ {T}_{3}\succ {T}_{4}\succ {T}_{2}$
9	$S\left({m}_{1}\right)=0.4020$, $S\left({m}_{2}\right)=0.3005$, $S\left({m}_{3}\right)=0.3155$, $S\left({m}_{4}\right)=0.3026$,$S\left({m}_{5}\right)=0.3521$	${T}_{1}\succ {T}_{5}\succ {T}_{3}\succ {T}_{4}\succ {T}_{2}$
10	$S\left({m}_{1}\right)=0.4056$, $S\left({m}_{2}\right)=0.3082$, $S\left({m}_{3}\right)=0.3278$, $S\left({m}_{4}\right)=0.3080$,$S\left({m}_{5}\right)=0.3561$	${T}_{1}\succ {T}_{5}\succ {T}_{3}\succ {T}_{2}\succ {T}_{4}$

Table 6 above presents the preference value ${m}_{i} (i = 1, 2, \dots ,5)$ of the companies ${T}_{i}(i = 1, 2, 3, \dots , 5)$ for various values of α using the GIFWGA, with respect to the criteria ${C}_{j}(j = 1, 2, \dots , 4)$.

**Table 6:** Preference value ${m}_{i} (i = 1, 2, \dots ,5)$ by using GIFWGA.

α	Preference value ${{\varvec{m}}}_{{\varvec{i}}}({\varvec{i}}=1,2,\dots ,5)$ by using the GIFWGA
2	${m}_{1}=\left(\mathrm{0.5569,0.5265}\right), {m}_{2}=\left(0.3150, 0.6867\right), {m}_{3}=\left(0.3912, 0.7640\right),$ ${m}_{4}=\left(0.4838, 0.5563\right) and {m}_{5}=\left(0.4459, 0.4785\right)$
3	${m}_{1}=\left(\mathrm{0.5569,0.5414}\right), {m}_{2}=\left(0.3150, 0.7054\right), {m}_{3}=\left(0.3912, 0.7791\right),$ ${m}_{4}=\left(0.4838, 0.5684\right) and {m}_{5}=\left(0.4459, 0.5113\right)$
4	${m}_{1}=\left(\mathrm{0.5569,0.5523}\right), {m}_{2}=\left(0.3150, 0.7225\right), {m}_{3}=\left(0.3912, 0.7921\right),$ ${m}_{4}=\left(0.4838, 0.5791\right) and {m}_{5}=\left(0.4459, 0.5400\right)$
5	${m}_{1}=\left(\mathrm{0.5569,0.5602}\right), {m}_{2}=\left(0.3150, 0.7378\right), {m}_{3}=\left(0.3912, 0.8030\right),$ ${m}_{4}=\left(0.4838, 0.5883\right) and {m}_{5}=\left(0.4459, 0.5646\right)$
6	${m}_{1}=\left(\mathrm{0.5569,0.5661}\right), {m}_{2}=\left(0.3150, 0.7512\right), {m}_{3}=\left(0.3912, 0.8121\right),$ ${m}_{4}=\left(0.4838, ,0.5962\right) and {m}_{5}=\left(\mathrm{0.4459,0.5857}\right)$
7	${m}_{1}=\left(\mathrm{0.5569,0.5706}\right), {m}_{2}=\left(0.3150, 0.7628\right), {m}_{3}=\left(0.3912, 0.8197\right),$ ${m}_{4}=\left(0.4838, 0.6029\right) and {m}_{5}=\left(\mathrm{0.4459,0.6038}\right)$
8	${m}_{1}=\left(\mathrm{0.5569,0.5740}\right), {m}_{2}=\left(0.3150, 0.7730\right), {m}_{3}=\left(0.3912, 0.8261\right),$ ${m}_{4}=\left(\mathrm{0.4838,0.6088}\right) and {m}_{5}=\left(\mathrm{0.4459,0.6196}\right)$
9	${m}_{1}=\left(\mathrm{0.5569,0.5768}\right), {m}_{2}=\left(\mathrm{0.3150,0.7818} \right), {m}_{3}=\left(0.3912, 0.8316\right),$ ${m}_{4}=\left(\mathrm{0.4838,0.6141}\right) and {m}_{5}=\left(\mathrm{0.4459,0.6333}\right)$
10	${m}_{1}=\left(\mathrm{0.5569,0.5790}\right), {m}_{2}=\left(0.3150, 0.7896\right), {m}_{3}=\left(0.3912, 0.8363\right),$ ${m}_{4}=\left(\mathrm{0.4838,0.6187}\right) and {m}_{5}=\left(\mathrm{0.4459,0.6453}\right)$

The ranking order and score function outcomes for the alternatives ${T}_{i}(i = 1, 2, 3, \dots , 5)$ have been assessed using the GIFWGA for the corresponding values ${{\text{m}}}_{{\text{i}}} (\mathrm{i }= 1, 2, \dots ,5)$. The results are shown above in Table 7 for various values of α.

**Table 7:** score functions ${\text{S}}({{\text{m}}}_{{\text{i}}})$ by using GIFWGA.

α	Score functions $\mathbf{S}({\mathbf{m}}_{\mathbf{i}})$	Ranking orders by using GIFWGA
2	$S\left({m}_{1}\right)=0.0908$, $S\left({m}_{2}\right)=-0.3051$ , $S\left({m}_{3}\right)=-0.3281$ , $S\left({m}_{4}\right)=-0.1373$ ,$S\left({m}_{5}\right)=-0.0868$	${T}_{1}\succ {T}_{5}\succ {T}_{4}\succ {T}_{2}\succ {T}_{3}$
3	$S\left({m}_{1}\right)=0.1207$, $S\left({m}_{2}\right)=-0.3419$ , $S\left({m}_{3}\right)=-0.3724$ , $S\left({m}_{4}\right)=-0.2065$ ,$S\left({m}_{5}\right)=-0.1779$	${T}_{1}\succ {T}_{5}\succ {T}_{4}\succ {T}_{2}\succ {T}_{3}$
4	$S\left({m}_{1}\right)=0.1187$, $S\left({m}_{2}\right)=-0.3579$ , $S\left({m}_{3}\right)=-0.3900$ , $S\left({m}_{4}\right)=-0.2450$ ,$S\left({m}_{5}\right)=-0.2309$	${T}_{1}\succ {T}_{5}\succ {T}_{4}\succ {T}_{2}\succ {T}_{3}$
5	$S\left({m}_{1}\right)=-0.1380$, $S\left({m}_{2}\right)=-0.3678$ , $S\left({m}_{3}\right)=-0.3993$ , $S\left({m}_{4}\right)=-0.2677$ ,$S\left({m}_{5}\right)=-0.2623$	${T}_{1}\succ {T}_{5}\succ {T}_{4}\succ {T}_{2}\succ {T}_{3}$
6	$S\left({m}_{1}\right)=-0.1906$, $S\left({m}_{2}\right)=-0.3752$, $S\left({m}_{3}\right)=-0.4052$, $S\left({m}_{4}\right)=-0.2818$,$S\left({m}_{5}\right)=-0.2825$	${T}_{1}\succ {T}_{4}\succ {T}_{5}\succ {T}_{2}\succ {T}_{3}$
7	$S\left({m}_{1}\right)=-0.2187$, $S\left({m}_{2}\right)=-0.3813$, $S\left({m}_{3}\right)=-0.4095$, $S\left({m}_{4}\right)=-0.2913$,$S\left({m}_{5}\right)=-0.2965$	${T}_{1}\succ {T}_{4}\succ {T}_{5}\succ {T}_{2}\succ {T}_{3}$
8	$S\left({m}_{1}\right)=-0.2368$, $S\left({m}_{2}\right)=-0.3864$, $S\left({m}_{3}\right)=-0.4129$, $S\left({m}_{4}\right)=-0.2979$,$S\left({m}_{5}\right)=-0.3069$	${T}_{1}\succ {T}_{4}\succ {T}_{5}\succ {T}_{2}\succ {T}_{3}$
9	$S\left({m}_{1}\right)=-0.2494$, $S\left({m}_{2}\right)=-0.3909$, $S\left({m}_{3}\right)=-0.4157$, $S\left({m}_{4}\right)=-0.3028$,$S\left({m}_{5}\right)=-0.3151$	${T}_{1}\succ {T}_{4}\succ {T}_{5}\succ {T}_{2}\succ {T}_{3}$
10	$S\left({m}_{1}\right)=-0.2585$, $S\left({m}_{2}\right)=-0.3948$, $S\left({m}_{3}\right)=-0.4181$, $S\left({m}_{4}\right)=-0.3066$,$S\left({m}_{5}\right)=-0.3218$	${T}_{1}\succ {T}_{4}\succ {T}_{5}\succ {T}_{2}\succ {T}_{3}$

The ranking orders yielded by the GIFWAA and GIFWGA operators across varying α values exhibit remarkable consistency in this decision-making context, supported by the findings presented in Tables 5 and 7. The decision-making results from Proposed MCDM in Tech Industry Evaluation for Startup Success underscore that although different α values may exert some influence on ranking orders, but the optimal choice remains unchanged.

**Figure 3:**
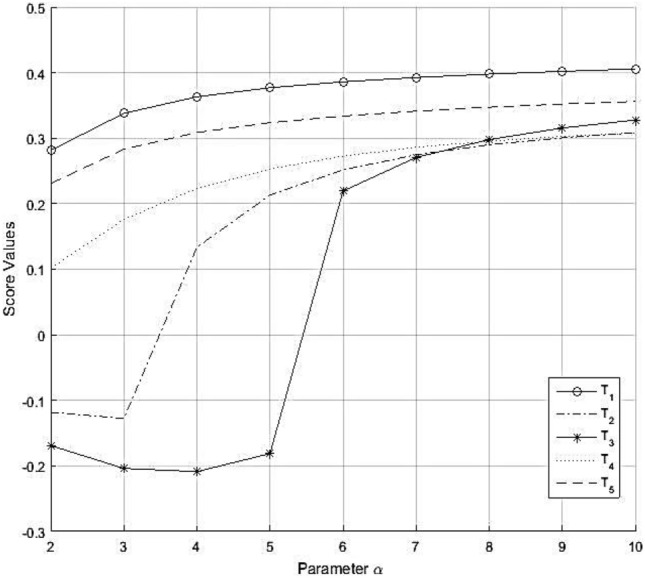
Score of the alternatives ${T}_{i}(i = 1, 2, 3, \dots , 5)$ based on the GIFWAA $\alpha \in [\mathrm{2,10}]$.

By utilizing the GIFWAA operator, the resulting score values exhibit variations corresponding to distinct values of the parameter $\alpha$ for the alternatives ${{\text{T}}}_{{\text{i}}}$ $(i=\mathrm{1,2},\mathrm{3,4},5).$ From the graph above in Fig. 3, showing score values with corresponding $\alpha$ values ranging from 2 to 10, it can be observed that ${T}_{1}$ and ${T}_{5}$ consistently maintain their positions in 1st and 2nd place. However, at $a = 8$ and onwards there is a change in the ranking orders of ${T}_{2}$, ${T}_{3}$ and ${T}_{4}$.

**Figure 4:**
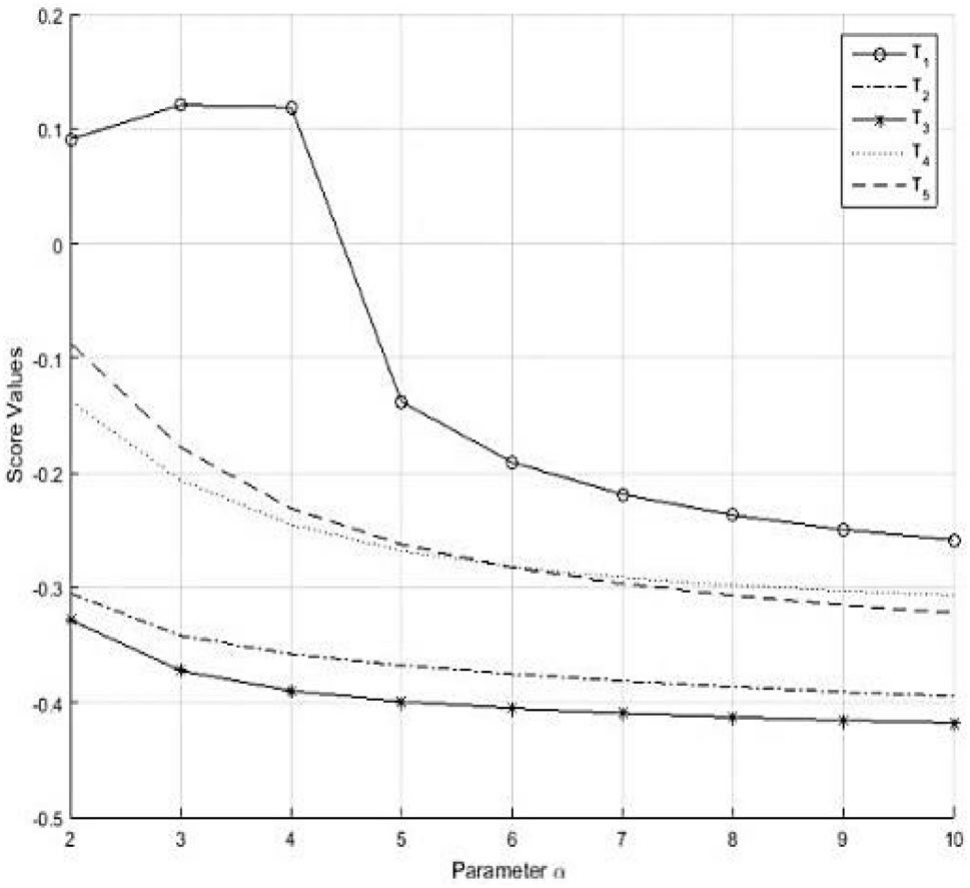
Score of the alternatives ${T}_{i}(i = 1, 2, 3, \dots , 5)$ based on the GIFWGA, $\alpha \in [\mathrm{2,10}]$.

Through application of the GIFWGA operator, the obtained score values show differences that correlate to different values of the parameter $\alpha$ for the alternatives ${T}_{i}$ $(i=\mathrm{1,2},\mathrm{3,4},5)$. As can be seen from the graph in Fig. 4 above, which displays score values with corresponding α values ranging from 2 to 10, ${T}_{1}$, ${T}_{2}$ and ${T}_{3}$ continuously hold their ranks in first, fourth, and fifth place. However, at $a = 6$, ${T}_{4}$ ${T}_{5}$ changed their positions from 3rd to 2nd place respectively, and they maintain these positions after $\alpha = 8$.

### Advantages

The following list includes the developed methodology's advantages.

- The decision-maker has a more flexible framework to express uncertain information in the model that is being presented since decision-making information is expressed using GIFNs.
- Traditionally, decision-makers frequently allocate different weights indiscriminately, ignoring uncertainty. We can mitigate the impact of unfair arguments on the decision outcomes in our proposed model by employing GIFOWGA or GIFOWAA, which weight arguments with small values. Essentially, we can use weights derived from the normal distribution based method^42^ to address data bias.
- Generalized Intuitionistic Fuzzy Aggregation Operators are incorporated into our model to combine data for every alternate. Because Generalized operators are more comprehensive versions of different existing operators, this increases the flexibility of the information aggregation process. As such, decision makers are free to choose particular variants of Generalized Intuitionistic Fuzzy Operators for data fusion based on personal preferences.

### Comparison

We evaluated our proposed model's validity and effectiveness by contrasting its results with those of several other operators that are currently in use.

#### Comparison with aggregation operator-based methods

Rather than using the matrix ${\varvec{Q}}={({{\varvec{m}}}_{{\varvec{i}}{\varvec{j}}})}_{5\times 4}$ in Table 3, we could use the decision matrix shown in Table 8 with the corresponding weight vector ${{\varvec{w}}}_{{\varvec{j}}}=\boldsymbol{ }{(0.192,\boldsymbol{ }0.205,\boldsymbol{ }0.192,\boldsymbol{ }0.222,\boldsymbol{ }0.189)}^{{\varvec{T}}}$. Subsequently, Table 9 below compares the previously used aggregation operators with the proposed operator for the data in Table 8.

**Table 8:** Generalized intuitionistic fuzzy decision matrix (GIFDM) (Adapted from^43^).

	**C**_**1**_	**C**_**2**_	**C**_**3**_	**C**_**4**_	**C**_**5**_
${\varvec{T}}$_1_	$\left(\mathrm{0.8,0.5}\right)$	$\left(\mathrm{0.6,0.9}\right)$	$\left(\mathrm{0.7,0.21}\right)$	$\left(\mathrm{0.8,0.7}\right)$	$\left(\mathrm{0.66,0.7}\right)$
${\varvec{T}}$_2_	$\left(\mathrm{0.9,0.5}\right)$	$\left(\mathrm{0.8,0.2}\right)$	$\left(\mathrm{0.6,0.4}\right)$	$\left(\mathrm{0.66,0.9}\right)$	$\left(\mathrm{0.70,0.41}\right)$
${\varvec{T}}$_3_	$\left(\mathrm{0.5,0.5}\right)$	$\left(\mathrm{0.6,0.7}\right)$	$\left(\mathrm{0.8,0.8}\right)$	$\left(\mathrm{0.77,0.32}\right)$	$\left(\mathrm{0.20,0.70}\right)$
${\varvec{T}}$_4_	$\left(\mathrm{0.35,0.81}\right)$	$\left(\mathrm{0.6,0.6}\right)$	$\left(\mathrm{0.72,0.5}\right)$	$\left(\mathrm{0.79,0.31}\right)$	$\left(\mathrm{0.72,0.65}\right)$

**Table 9:** Alternative rankings using some weighted aggregation operators ($\boldsymbol{\alpha }=4$ ).

Aggregation operators		Score values	Ranking	Optimal alternate
^8^	IFWA	Not possible	No	No
^8^	EIFWA	Not possible	No	No
^45^	PFWA	Not possible	No	No
^46^	PFEWA	Not possible	No	No
^31^	IFWG	Not possible	No	No
^44^	EIFWG	Not possible	No	No
^45^	PFWG	Not possible	No	No
^47^	PFEWG	Not possible	No	No
^48^	q-ROFWA	${\text{S}}\left({{\text{m}}}_{1}\right)$= 0.1972, ${\text{S}}\left({{\text{m}}}_{2}\right)$= 0.3188, ${\text{S}}\left({{\text{m}}}_{3}\right)$= 0.1097,${\text{S}}\left({{\text{m}}}_{4}\right)=0.1452$	${T}_{2}\succ {T}_{1}\succ {T}_{4}\succ {T}_{3}$	${T}_{2}$
^48^	q-ROFEWA	$S\left({m}_{1}\right)$= 0.1834, $S\left({m}_{2}\right)$= 0.3009, $S\left({m}_{3}\right)$= 0.0939,$S\left({m}_{4}\right)=0.1347$	${T}_{2}\succ {T}_{1}\succ {T}_{4}\succ {T}_{3}$	${T}_{2}$
Proposed	GIFWAA	$S\left({m}_{1}\right)=0.3332$*, *$S\left({m}_{2}\right)=0.3757$* , *$S\left({m}_{3}\right)=0.2877$, $S\left({m}_{4}\right)=0.3087$	${T}_{2}\succ {T}_{1}\succ {T}_{4}\succ {T}_{3}$	${T}_{2}$
Proposed	GIFWAG	$S\left({m}_{1}\right)=-0.2221$*, *$S\left({m}_{2}\right)=0.2302$*, *$S\left({m}_{3}\right)=-0.2969$ , $S\left({m}_{4}\right)=-0.2029$	${T}_{2}\succ {T}_{4}\succ {T}_{1}\succ {T}_{3}$	${T}_{2}$

The IFWA^8^, IFWG^31^, EIFWA^8^ and EIFWG^44^ have a simple calculation process, but their application is limited. Only decisions involving information expressed as an intuitionistic fuzzy number (IFN) can be handled by it. IFWA, IFWG, EIFWA and EIFWG operators are not sufficient to handle our proposed MCDM problem because it includes an evaluation value that goes beyond the IFN. Currently, the evaluation information must be elicited as a PFN in order to use the PFWA^45^, PFWG^45^, PFEWA^46^ and PFEWG^47^ operators. But in PFN, the squared sum of MD and NMD is restricted to one. Therefore, the MCDM problem given in Table 8 cannot be solved using the PFWA, PFWG, PFEWA and PFEWG operators. The suggested model can now be handled by GIFWAA, GIFWGA, GIFOWAA and GIFOWGA operator because the parameter $\boldsymbol{\alpha }$ increases the adaptability of the aggregation process. Increasing the parameter $\boldsymbol{\alpha }$ will increase the information's range. Consequently, we can state that the models based on IFWA, IFWG, EIFWA, EIFWG, PFWA, PFWG, PFEWA and PFEWG operators perform worse than our suggested model. All of the alternatives' appraisal scores are now calculated with the GIFWAA and GIFWGA operator. Next, the options are arranged according to score value in descending order. The outcomes of the ranking are shown in Tables 9. Tables 9 show that the best option is always ${{\varvec{T}}}_{2}$, which can be obtained by using either of the operators, GIFWGA or GIFWAA.

It is evident from Table 9 that the problem in Table 8 cannot be solved by using IFWA, IFWG, EIFWA and EIFWG operators, and PFWA, PFWG, PFEWA and PFEWG Aggregation Operators because they are only capable of handling Pythagorean fuzzy information and IF information. By using the GIFWGA and GIFWAA operators, on the other hand, it is found that while the alternatives' score values differ, their ranking order stays the same, and ${{\varvec{T}}}_{2}$ ends up being the best option. The suggested method is more effective than traditional methods since it can be tailored to elicit fuzzy data over a wider range. Consequently, our proposed Multi Criteria Decision Making (MCDM) approach is more suitable for GIFNs and more generalized.

Although the suggested model can also be handled by q-ROFWA, q-ROFEWA, q-ROFFWA, and Yq-ROFWA operators, but these operators are sub case of our proposed GIFWAA operator as explained in the introduction. Also if we have intuitionistic fuzzy set of root type i.e a data of the form in which half of the sum of the square root of MD and NMD less or equal to one, then q-ROFWA, q-ROFEWA, q-ROFFWA, and Yq-ROFWA operators are fail to handle it.

## Conclusion

This research introduces several aggregation operators on GIFS_B_s. GIFWAA operator, GIFWGA operator, GIFOWAA operator, and GIFOWGA operator, each with their respective desired properties. Two approaches have been proposed to address multi-criteria decision-making problems in the GIFS_B_ environment using these operators. The first approach employs GIFWAA operators, while the second approach utilizes GIFWGA operators, providing decision-makers with multiple options for evaluating decisions. These approaches have been implemented using various values of α, showcasing the versatility of the proposed operators.

Furthermore, the operators introduced in this study offer distinct advantages compared to their predecessors. Generalized Intuitionistic Fuzzy Sets (GIFS_B_s) exhibit a unique property: for different values of $\alpha$ ,they encompass Atanassov's intuitionistic fuzzy set, root type intuitionistic fuzzy sets, and intuitionistic fuzzy sets of the second type as specific cases, thus demonstrating that GIFS_B_s constitute a generalization of these well-established constructs. Consequently, the operators defined on GIFS_B_s exhibit a broader scope and applicability compared to those defined on traditional IFSs. Notably, the operators defined in^8^ and^41^ are special cases of the operators defined in this research for $\alpha =1$ and $\alpha =2$. It turns out that the GIFS_B_ concept is a very adaptable method that addresses a wide range of specific scenarios of current IFS extensions, in addition to solving the previously mentioned issues. Although this idea can be expressed in many ways, α parameterizes MD and NMD in the formal definition. The standard IFS is used again when α equals 1. It becomes Pythagorean FS (PFS)^24^ or IFS 2-type (IFS2T)^22^ when α equals 2. When α = 3, it represents Fermatean FS (FFS)^26^. α = n for any positive integer n yields IFS n-type (IFS-nT), generalized orthopair FS, or q-rung orthopair fuzzy sets^23^. Moreover, when α is set to 1/2, it simplifies to the IFS root type (IFSRT)^21^.

This inclusive characteristic significantly enhances the relevance and versatility of the operators, making them a more comprehensive approach to addressing complex decision-making challenges.

Future research will focus on introducing the aggregation operators on GIFS_B_s to address some more complicated problems in dynamic environment.

## Data Availability

All data generated or analysed during this study are included in this published article (and its Supplementary Information files).
